# Antibacterial potential, DNA binding and molecular docking investigations of newly green synthesized zinc oxide/chitosan/vancomycin nanocomposite using *Bacillus licheniformis* ATCC 4527 against some drug-resistant bacteria

**DOI:** 10.1186/s12934-026-02928-9

**Published:** 2026-02-11

**Authors:** Shimaa M. El-Salamony, Zakaria A. M. Baka, Mohamed I. Abou-Dobara, Hanaa M. Salama, Mohamed M. El-Zahed

**Affiliations:** 1 Botany and Microbiology Department, Faculty of Science, Damietta University, New Damietta, Egypt; 2 Chemistry Department, Faculty of Science, Port Said University, Port Said, Egypt

**Keywords:** Green synthesis, ZnO, Nanocomposite, *Bacilluslicheniformis*, Antibacterial, Multidrug resistance

## Abstract

**Background:**

The increasing crisis of multidrug-resistant (MDR) bacteria requires the creation of new, highly effective, and safe antibacterial agents. This study introduces a simple and cost-effective green synthesis method for a novel zinc oxide/chitosan/vancomycin (ZnO/CS/VA) nanocomposite to improve antibacterial activity against priority MDR strains. Zinc oxide nanoparticles (ZnO NPs) were biosynthesized using *Bacillus licheniformis* ATCC 4527 and then combined with vancomycin (VA) and chitosan (CS) in an environmentally friendly process. Transmission electron microscopy (TEM), Fourier transform infrared spectroscopy (FTIR), X-ray diffraction (XRD), ultraviolet-visible spectroscopy (UV-Vis), and zeta potential studies were conducted to validate and characterize the production of ZnO/CS/VA. The antibacterial activity of the nanoparticles was tested against several multidrug-resistant (MDR) strains, including *Bacillus cereus* HES3, methicillin-resistant *S. aureus* (MRSA), *Escherichia coli* D8, *Pseudomonas aeruginosa*, and *Klebsiella pneumoniae* H4. Further assessment included molecular docking interactions with *E. coli* (PDB: 3T88) and MRSA (PDB: 4DKI) targets, CT-DNA binding, and cytotoxicity testing on the Vero cell line. Characterization confirmed the successful formation of the nanocomposite, revealing ZnO nanoparticles ranging from 33.24 to 69.11 nm and high stability (zeta potential: -17.78 mV). The ZnO/CS/VA nanocomposite exhibited superior dose-dependent antibacterial activity compared to ZnO nanoparticles or VA alone against all tested MDR strains, including MRSA and *E. coli* D8. Molecular docking demonstrated strong binding, with scores ranging from -0.7 to -10.85 kcal/mol. The nanocomposite exhibited a hypochromism when interacting with CT-DNA, suggesting intercalation. Importantly, the CC_50_ values of ZnO NPs and ZnO/CS/VA against Vero cells were 146.62±1.03 and 162.86±1.07 μg/mL, respectively, indicating their high level of safety.

**Conclusions:**

The green-synthesized ZnO/CS/VA nanocomposite is a promising alternative candidate for treating various MDR bacterial infections. It offers utility in biomedical and pharmaceutical applications due to its high efficacy and low cytotoxicity.

**Supplementary Information:**

The online version contains supplementary material available at 10.1186/s12934-026-02928-9.

## Background

Since the natural genetic evolution of antibiotic resistance has reached to unprecedented levels in the twenty-first century, antimicrobial resistance (AMR) has become a critical global health concern that necessitates early intervention [1]. Bacterial resistance to antibiotics limits their effectiveness in healthcare, and there is compelling evidence that antibiotic misuse will ultimately lead to the development of resistance [2]. Inappropriate use of antibiotics could prevent the colony from being completely destroyed, leading to the formation of antibiotic-resistant bacteria. Additionally, the Food and Drug Administration (FDA) emphasizes that sharing antibiotics, improper storage, and skipping doses can contribute to the development of antibiotic-resistant bacteria [3]. Recently, infections have been persisting and spreading, while conventional antibiotic treatments have proven to be relatively ineffective [4]. In 2019, it is predicted that drug-resistant diseases will cause an astonishing 4.95 million deaths worldwide, with the majority of the clinical burden falling on low- and middle-income countries (LMICs), especially in Sub-Saharan Africa [5]. By 2050, it is predicted that AMR will kill 10 million people annually if no action is taking [6, 7].

There are three recognized categories of AMR: multidrug resistance (MDR), extended drug resistance (XDR), and complete drug resistance (TDR). XDR is defined as not being susceptible to at least one agent in all but two antimicrobial classes; MDR is defined as acquired lack of susceptibility to at least one agent in three or more antimicrobial classes. TDR is defined as not being susceptible to all agents in all available antimicrobial classes [8]. In order to survive in the presence of an antibiotic, bacterial strains must be capable of disrupting one or more of the critical functions necessary for the antimicrobial drug to work [9, 10]. Bacterial species have four basic methods for survival: (i) preventing the antibiotic from reaching its target by decreasing its ability to enter the bacterial cell; (ii) eliminating antibacterial medications from the cell through the efflux pump mechanism; (iii) undergoing changes or deterioration that make an antibiotic ineffective; and (iv) making changes or adjustments to the antibacterial target of the bacteria. *Klebsiella pneumoniae* and *Pseudomonas aeruginosa*, two MDR bacteria, use various strategies to resist different classes of antibiotics such as carbapenems, cephalosporins, aminoglycosides, and fosfomycin [11, 12]. Some of these strategies include increased efflux, drug inactivation, or altered binding to the target site, all of which can result in resistance. In addition, resistance is exacerbated by the production of extended-spectrum beta-lactamases (ESBL) or the formation of biofilms by numerous *K. pneumoniae* and *P. aeruginosa* strains [13]. Other bacteria, such as *Escherichia coli* and *Bacillus cereus*, have also been shown to develop resistance to antimicrobial drugs. This can occur either by acquiring plasmids that harbor resistance genes or by undergoing chromosomal gene alterations [14].

The glycopeptide antibiotic vancomycin (VA) prevents the production of peptidoglycan in the bacterial cell wall. It is used to treat lung infections such as bronchitis and pneumonia, skin infections, bone infections, enterocolitis, and endocarditis due to its potent bactericidal action against various resistant bacterial strains [15]. Recently, methicillin-resistant *S. aureus* (MRSA) and MDR mycobacteria have shown a resistant behavior that can persist in intracellular sites after medication therapy [16]. Several studies on VA-loaded nanoparticles (NPs) have been reported [17–19].

Nanotechnology is a rapidly growing field with applications in numerous scientific and research fields. The aim is to produce novel chemicals and molecules at the nanoscale level that may have promised antimicrobial potential to treat resistant strains [20–23]. A “nano” is a unit of measurement equal to one billionth of a metre, or 10^− 9^. The term “nanoparticles” refers to solid particles, particulate dispersions, or assemblies of atoms that measure between one and one hundred nanometers in size. The Greek term “dwarf,” which implies something incredibly small, is the origin of the word “nano”. Nanoparticles (NPs) have many uses due to their unique characteristics. NPs vary in size, shape, and dimension, as well as in the material they are made of. The potential applications of this material are determined by its chemical properties [24]. These properties include the stability and sensitivity of the NPs to different environmental conditions such as heat, light, moisture, and the environment, as well as their reactivity with the target. NPs can be used in biological and environmental applications because of their antibacterial, antifungal, disinfecting, and poisonous qualities [25–30]. NPs without carbon atoms are referred to as inorganic. NPs made of metal or metal oxide are known as inorganic NPs. Metal oxide NPs such as zinc oxide nanoparticles (ZnO NPs) have promising applications in biology, including diagnosis and therapy. These NPs have a wide range of applications, including their mechanical, regenerative, and antimicrobial qualities [31–34]. The molecular weight of ZnO NPs is 81.38 g/mol, and they are odorless, white powders. According to the FDA, it is a drug that is generally recognized as safe (GRAS) compared to other inorganic NPs [35].

Nanomaterials can be synthesized in two different ways. The bottom-up, or self-assembly, method of nanofabrication applies physical or chemical forces acting at the nanoscale to combine building blocks into functional structures [36]. A bottom-up approach is utilized to create materials, starting from the atomic level and advancing to the nanoparticle level. Chemical vapor deposition (CVD), pyrolysis, spinning, sol-gel, and biosynthesis among the most commonly used bottom-up techniques for producing NPs [37]. Biological systems serve as an inspiration for bottom-up methods, as they utilize chemical forces to construct all the components necessary for life [38]. The top-down, or destructive, technique disassembles a material into its atomic components. This method can break large amounts of material into tiny, nanoscale pieces. While top-down methods are simpler to employ, they are not as effective for producing particles with complex shapes or sizes [39]. One of the primary disadvantages of this technology is the challenge it presents in producing particles of the right size and shape. Microbes can create inorganic chemicals intracellularly or extracellularly, often at nanoscale with desired and distinguished shapes [40]. The primary mechanisms of microbial resistance to these metals include chemical detoxification of most toxic heavy metals and energy-dependent ion efflux from the cell. This process is facilitated by membrane proteins that function as ATPase, chemiosmotic cation, or proton anti-transporters [41].

Probiotics are beneficial bacteria that, when consumed in sufficient quantities, can improve the host’s health. Numerous studies have shown that probiotics have a variety of positive health effects, including preventing allergic reactions, enhancing intestinal immune function, managing gastrointestinal disorders, protecting the heart, and exhibiting antioxidative and antitumorigenic properties. Additionally, probiotics have been found to have a hypocholesterolemic effect [42, 43].

*Bacillus licheniformis* is a distinct species within the *Bacillus* genus known for its production of a diverse range of antibiotic compounds. This bacterium has the potential to be utilized as a probiotic in the treatment of dysbacteriosis, a condition resulting from various diseases [44]. The benefits of *B. licheniformis* as a probiotic are associated with its ability to produce a wide range of substances with antibacterial, antioxidant, and immunomodulatory properties [45, 46]. Abinaya et al. [47]. synthesized ZnO NPs in a new and effective manner by using the exopolysaccharides (EPS) from the probiotic strain *B. licheniformis* Dahb1. Gomaa [43] reported that microorganisms produce and release proteins and enzymes during biosynthesis processes that can stabilize particles and reduce the amount of metal ions. According to Tripathi et al. [49]. , ZnO NPs can be stabilized by enzymes produced by *B. licheniformis* bacterial cells [50].

ZnO NPs have been utilized in various agricultural, industrial, and medical fields because of their capability to negatively impact the intracellular chemical constituents, damage microbial DNA or alter microenvironments near microorganisms, locally generating reactive oxygen species (ROS) that demonstrate their antimicrobial activity [51, 52]. This can result in interactions with the -SH group of the bacteria’s enzymes and dysfunction of organelles, leading to denaturation of proteins and DNA damage. Ultimately, this disrupts the transcription and replication of microbial DNA, leading to cell death [53]. Based on these findings, several recent studies have focused on fabricating novel drugs that incorporate metal or metal oxide NPs as nanocomposites [54–59]. These drugs could possess potential and effective antimicrobial activities. Another potential antibacterial mechanism involves the displacement of magnesium ions, which disrupts bacterial metabolism, and the release of H_2_O_2_ [60].

On the other hand, efflux pumps, electrostatic repulsion, biofilms and other extracellular polymeric materials, enzyme detoxification, volatilization, and genetic alterations have all been used to demonstrate microbial adaptability to NPs [61, 62]. Additionally, natural environments can also lead to changes in NPs [63]. Therefore, it is important to explore the potential of different biogenic metal NPs or their combinations for treating drug-resistant microbes [64–67]. The current study aimed to create new nanocomposite by combining ZnO NPs and VA with chitosan (CS) and to investigate the antibacterial potential and ability to cleave DNA against drug-resistant bacterial strains. The current study hypothesizes that combining the distinct antimicrobial mechanisms of action will result in superior synergy. ZnO NPs target bacteria through ROS generation and physical damage [51]. The cationic nature of CS promotes membrane disruption and entry [68], while VA maintains targeted inhibition of cell wall synthesis, especially against Gram-positive strains [69]. This combined approach is designed to circumvent resistance pathways more effectively than any single component. CS serves as an ideal, biocompatible polymeric matrix, utilizing its mucoadhesive and structural properties to stabilize the ZnO NPs and function as a nanocarrier for VA to enhanced drug delivery and targeting [70]. The incorporation of CS is crucial for maintaining the system’s physical integrity and safety. CS acts as a green stabilizing agent to prevent the undesirable agglomeration of ZnO NPs, maintaining their high surface area and reactivity. This system is expected to increase the local concentration of VA at the infection site, improving therapeutic efficacy while potentially reducing the systemic toxicity associated with high-dose free VA administration [71].

Multi-modal nature of the zinc oxide/chitosan/vancomycin (ZnO/CS/VA) nanocomposite is essential to understand the nanocomposite’s interaction with target bacterial proteins, enzymes, DNA, and other structural compounds and calculate the binding affinities thereby providing molecular-level support for the observed synergistic antibacterial efficacy. In silico methods have become valuable scientific tools for exploring and elucidating biological mechanisms [72, 73]. Among these methods, molecular docking is widely employed to assess the suitability of potential drug candidates prior to experimental testing. Today, computer-aided drug design relies heavily on docking techniques to identify novel therapeutic agents. Molecular docking also plays a key role in drug discovery and development by analyzing drug–receptor interactions and predicting the binding activity and affinity of ligands toward target protein sites [74, 75]. Thus, the primary objective of this study is to synthesize, characterize, and evaluate the synergistic antimicrobial efficacy and molecular mechanism via docking to predict the binding affinity between the prepared nanocomposite and the receptors 3T88 and 4DKI, which correspond to *E. coli* and MRSA, respectively. Additionally, we will investigate the DNA binding, and safety profile of the novel ZnO/CS/VA nanocomposite against a panel of clinically relevant MDR pathogens.

## Materials and methods

### Bacterial strains

*Bacillus licheniformis* ATCC 4527, *B. cereus* HES3 (Accession: OR553494), MRSA ATCC 43,300, *E. coli* D8 (Accession: MF062579), *K. pneumoniae* H4 (Accession: OQ875950), and *P. aeruginosa* ATCC 27,853 were provided by the Microbiology Lab at the Faculty of Science, Damietta University.

### Antibiotic susceptibility test

The antibiotic susceptibility test (AST) for bacterial strains was conducted using the disc diffusion technique in accordance with the Clinical and Laboratory Standards [76]. Cooled, melted Mueller-Hinton agar (MHA, Oxoid, UK) flasks were inoculated with 50 µL of each bacterial suspension (0.5 McFarland Standard; 1–2 × 10^8 CFU/mL) and then placed on sterile Petri dishes. Following solidification, sterile forceps were used to carefully load antibiotic discs representing different antibiotic classes: 30 µg/mL of ampicillin, 30 µg/mL of amoxicillin/clavulanate, 30 µg/mL of cefepime, 30 µg/mL of ceftriaxone, 30 µg/mL of chloramphenicol, 5 µg/mL of ciprofloxacin, 15 µg/mL of erythromycin, 10 µg/mL of gentamicin, 30 µg/mL of rifamycin, 5 µg/mL of levofloxacin, 10 µg/mL of tobramycin, and 30 µg/mL of vancomycin. They were then incubated for 24 h at 37 °C. The diameter of the inhibition zone was then measured and recorded.

### The cloverleaf test

The MHA plates were inoculated with 0.5 McFarland suspension of the tested bacterial strain using the spread plate method. Subsequently, the tested strains were streaked radially outward from a penicillin disc (10 U) positioned in the center of the plate, with each streak approximately 0.25 cm wide. The plates were then incubated at 37 °C for 18 h. If the isolate produces β-lactamase, it will exhibit a cloverleaf pattern [77].

### Masuda double-disc test

A 10 U penicillin disc was placed in the center of the MHA plates that had been swabbed with the tested strain. Additionally, filter paper discs were inoculated by immersing them in a 24-hour bacterial culture (0.5 MacFarland) and then positioned 10 mm away from the main disc. Positive β-lactamase synthesis is indicated by the lack of inhibition around the centrally inserted disc, as shown by the test discs after the incubation period [78].

### Extended-spectrum beta-lactamases detection

The bacterial strains were tested for possible ESBL production using ceftriaxone (CTR, 30 µg), cefotaxime (CTX, 30 µg), and cefoxitin (FOX, 30 µg). Isolates with reduced sensitivity to one or more of these drugs were considered to be ESBL-producing. Their zone of inhibition widths was (16 ± 0.3 mm) for FOX, (15 ± 0.2 mm) for CTX, and (18 ± 0.3 mm) for CTR [79]. Additionally, the Vitek 2 AST-N255 G-ve susceptibility card for ESBL detection was used to validate the results [80].

### Biogenic production of zinc oxide nanoparticles

*Bacillus licheniformis* ATCC 4527 was inoculated into 250 Erlenmeyer flasks containing 100 mL of nutrient broth for the biosynthesis investigation. The flasks were then incubated for 24 h at 37 °C and 150 rpm. After the incubation period, the culture was centrifuged for 20 min at 4000 rpm, and the cell biomass (CB) was separated for further analysis [81]. For the CB method, the biomass was suspended in a 3 mM solution of zinc nitrate solution (zinc nitrate hexahydrate, purum p.a., crystallized, ≥ 99.0% (KT), Sigma-Aldrich, USA) after being washed three times with 200 µL of distilled water. The reaction mixture was shaken at 37 °C and 150 rpm for 24 h under dark conditions. After incubation, the supernatant was removed from the reaction mixture using centrifugation at 4000 rpm for 15 min. Subsequently, the bacterial pellets were ultrasonicated and centrifuged at 4000 rpm for 5 min to eliminate bacterial cell residues. Finally, ZnO NPs were collected using centrifugation. The biogeneration process of NPs was measured by withdrawing 3 mL of the sample, and the absorbance was measured in the range of 200 to 600 nm at a resolution of 1 nm using a UV-visible spectrophotometer (JASCO, V-630) against the blank [82].

### Green synthesis of zinc oxide/chitosan/vancomycin nanocomposite

3 mg of ZnO NPs were dispersed in 10 mL of distilled water, and the mixture was then ultrasonically agitated for 15 min using an Elmasonic S100H ultrasonic bath (50/60 Hz, Germany). 3 mg of CS (MW 50–190 KDa, degree of deacetylation: ≥85%, Sigma-Aldrich, USA) was dissolved in 2.5% acetic acid (pH 8), and then mixed with an equal volume solution of 1:1 v/v ZnO NPs at room temperature. A magnetic stirrer/hot plate (Stuart UC152, UK) was used to stir the reaction mixture at 500 rpm for 20 min while adding 3 mg of antibiotic VA (Mylan Pharmaceuticals Ltd., Ireland). After 6 h, the drug-capped loading NPs were centrifuged at 5000 rpm, and the residue was washed three times using distilled water and water [83].

### Characterization of the prepared nanomaterials

A double beam UV–Vis spectrophotometer V-630 (JASCO, UK) and Fourier transform infrared spectroscopy (FTIR, FT/IR-4100typeA) were used to analyze the biogenesis of ZnO NPs. An X-ray diffractometer (model LabX XRD-6000, Shimadzu, Japan), a transmission electron microscope (TEM) apparatus (200 kV, TEM JEOL JEM-2100, Japan), and a zeta potential analyzer (Malvern Zetasizer Nano-ZS90, Malvern, UK) were used to study the properties of ZnO NPs.

### Antibacterial activity using agar well diffusion technique

Several bacterial models, including Gram-positive bacteria (*B. cereus* OR553494 and MRSA ATCC 25923), and Gram-negative bacteria (*E. coli* MF062579, *K. pneumoniae* OQ875950, *P. aeruginosa* ATCC 27853) were used to test the antibacterial properties of ZnO NPs and a zinc oxide/chitosan/vancomycin nanocomposite (ZnO/CS/VA). Mueller-Hinton agar (MHA) plates were prepared and inoculated with 0.5 McFarland of bacteria or yeast. A solution containing 150 µg/mL of ZnO NPs, CS, and ZnO/CS/VA in sterile distilled water was then added (100 µL) into wells (5 mm) in nutrient agar plates. VA was used as a standard antibacterial agent. The agar plates were incubated at 37 °C for 24 h. Zones of inhibition (ZOI) were observed following the incubation period [84].

### Minimum Inhibition concentration and minimum bactericidal concentration

The broth dilution method was utilized to determine the minimum inhibitory concentration (MIC) for the biosynthesized ZnO NPs and ZnO/CS/VA [85]. Over a period of 24 h, 100 µL of overnight bacterial cultures were inoculated into 50 ml of Mueller-Hinton broth medium (MHB, Oxoid, UK) that was supplemented with varying concentrations (0–100 µg/mL) of ZnO NPs, CS, VA, or ZnO/CS/VA. The cultures were incubated at 37 °C and 150 rpm for 24 h. MIC values (indicating no apparent growth) were measured at 600 nm using spectrophotometry. To determine the minimum bactericidal concentration (MBC), 10 µL aliquots of each set were inoculated on MHA plates and incubated for 24 h at 37 °C to measure the MBC. The MBC values (indicating no visible colonial growth on plates) were then noted.

### Calf thymus DNA binding study

Electronic absorption spectroscopy was utilized to study the binding characteristics of the produced nanocomposite to calf thymus DNA (CT-DNA). The stock solution of CT-DNA was prepared in a 5 mM Tris–HCl/50 mM NaCl buffer (pH = 7.2) with a UV absorbance ratio at 260 and 280 nm (A_260_/A_280_) of approximately 1.8–1.9. This indicates that the DNA was sufficiently free of protein [86]. The concentration of the stock solution of CT-DNA was determined using UV absorbance at 260 nm (Ɛ = 6600 M^− 1^ cm^− 1^) [87]. Using 1 cm quartz cuvettes at room temperature (25 °C), electronic absorption spectra were obtained by maintaining the nanocomposite concentration at 1.00 × 10^− 3^ mol/L and gradually increasing the CT-DNA concentration from 0.00 to 1.30 × 10^− 4^ mol/L. To eliminate the interference of CT-DNA itself, an equal amount of CT-DNA was added to both the chemical solutions and the reference buffer solution. The following formula was used to determine the nanocomposite’s intrinsic binding constant (K_b_) with CT-DNA:

$$\:\frac{\left[\mathrm{D}\mathrm{N}\mathrm{A}\right]}{({\upepsilon\:}\mathrm{a}-{\upepsilon\:}\mathrm{f})}=\frac{\left[\mathrm{D}\mathrm{N}\mathrm{A}\right]}{\:({\upepsilon\:}\mathrm{b}-{\upepsilon\:}\mathrm{f})}+\frac{1}{\mathrm{K}\mathrm{b}({\upepsilon\:}\mathrm{a}-{\upepsilon\:}\mathrm{f})}$$

Where [DNA] is the concentration of CT-DNA in base pairs, є_a_ is the molar extinction coefficient of the free compound in solution, and b is the molar extinction coefficient of the nanocomposite when fully bonded to DNA. Additionally, the molar extinction coefficient for the A_obs_/[nanocomposite] at the specified DNA concentration is denoted by є_b_. When [DNA]/(є_a_–є_f_) is plotted against [DNA], K_b_ is obtained by dividing the slope by the intercept.

### Molecular docking measurements

The docking simulations were carried out using MOE software. Protein structures (3T88 and 4DKI) were obtained from the PDB and then prepared by removing water molecules, adding hydrogen atoms, and energy minimization. The site finder identified active binding sites, which served as dummy sites for the binding pocket. Ligands (ZnO NPs and VA) were optimized using the MMFF94x force field to achieve a low-energy conformation.

ZnO nanocluster model (Zn₁₂O₁₂) was constructed using crystal data from the wurtzite phase (JCPDS card no. 36-1451). The geometry was optimized using the AMBER10:EHT force field implemented in MOE, with partial charges assigned via the extended Hückel method to maintain electrostatic neutrality [88–90]. Energy minimization was performed until the RMS gradient < 0.01 kcal/mol/Å. The minimized structure was imported into the MOE docking module as a rigid ligand for interaction analysis.

The triangle matcher technique was used to position ligands at the specified location, with the receptor atoms being docked for 100 nanoseconds. The GBVI/WSA dG procedures were then used for rescoring, with the London dG serving as the scoring function. For each ligand-protein pair, multiple poses were generated, and the top five were selected for further examination. 2D and 3D interaction diagrams were created to illustrate how the ligands bind to the active sites of each protein. These graphical representations are based on specific interactions. The docked complexes were examined to identify the interactions between the studied ligands and the active site residues of the protein.

### Cytotoxicity of ZnO NPs and ZnO/CS/VA

ZnO NPs and ZnO/CS/VA were tested for their cytotoxic effects on Vero cells (ATCC, Rockville, MD). Dulbecco’s Modified Eagle’s Medium (DMEM) was supplemented with 10% heat-inactivated fetal bovine serum, 1% L-glutamine, and 50 µg/ml gentamicin to support the growth of Vero cells. During the exponential growth phase, 1 × 10^4^ cells per well in 100 µl of growth media were plated into 96-well tissue culture plates using a multichannel pipette. Afterwards, the cells were given a full day to adhere. The corresponding wells were then treated with ZnO NPs or ZnO/CS/VA to achieve final concentrations ranging from 0 to 500 µg/ml. The number of live cells after a 24-hour period was counted using the MTT assay, as outlined by Mosmann [66]. The incubation took place in an incubator with 5% CO_2_ at 37 °C. The survival curve for each cell line after treatment with ZnO NPs or ZnO/CS/VA was determined by plotting the relationship between the concentration of these substances and the remaining cells.

### Statistical analysis

Software SPSS version 18 was utilized to perform statistical analysis on the data. After conducting a one-way analysis of variance (ANOVA) and Duncan’s multiple range test, the results of each experiment were presented as the mean ± standard deviation (SD). A significance level of *p* < 0.05 was applied [92].

## Results

### AST investigations

**Fig. 1:**
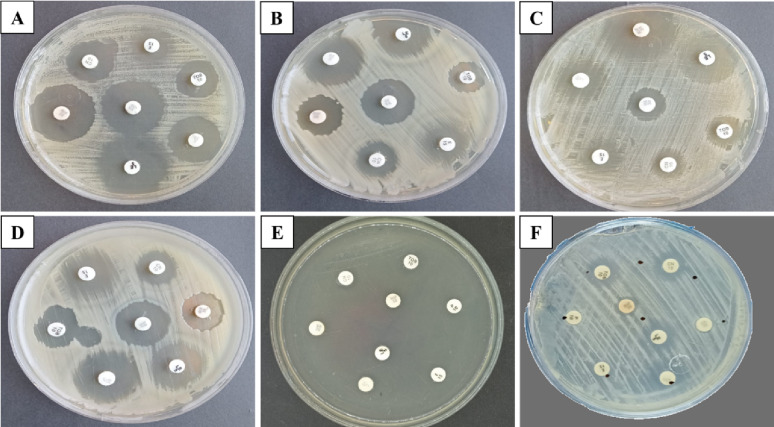
Antibiotic susceptibility test for *B. licheniformis*; **A**, *B. cereus* HES3; **B**, MRSA; **C**, *E. coli* D8; **D**, *P. aeruginosa*; **E**, and *K. pneumoniae* H4; **F**

Disc diffusion techniques were utilized to investigate the antibiotic susceptibility of the selected bacterial strains. Table 1; Fig. 1 present the strain’s susceptibility to all tested antibiotics. Chloramphenicol, gentamicin, tetracycline, and tobramycin exhibited the highest activity against those strains, with sensitivity ratio values of 88.33%, followed by levofloxacin and rifamycin at 66.67%. Next, an intermediate level of sensitivity was observed for cefepime, ciprofloxacin, erythromycin, and vancomycin (50%). *P. aeruginosa* exhibited the highest resistance pattern to all tested antibiotics, followed by *K. pneumoniae* H4 in comparison to other strains.

**Table 1:** Antibiotic sensitivity and resistance pattern of bacterial strains

Antibiotic	Concentration (µg/ml)	Antibiotics susceptibility	
		No. sensitive (%)	No. resistant (%)
Ampicillin	30	16.67	83.33
Amoxycillin/clavulanate	30/10	33.33	66.67
Cefepime	30	50	50
Ceftriaxone	30	33.33	66.67
Chloramphenicol	30	83.33	16.67
Ciprofloxacin	5	50	50
Cotrimoxazole	25	33.33	66.67
Erythromycin	15	50	50
Gentamicin	10	83.33	16.67
Levofloxacin	5	66.67	33.33
Rifamycin	30	66.67	33.33
Tetracycline	30	83.33	16.67
Tobramycin	10	83.33	16.67
Vancomycin	30	50	50

### The cloverleaf test

**Fig. 2:**
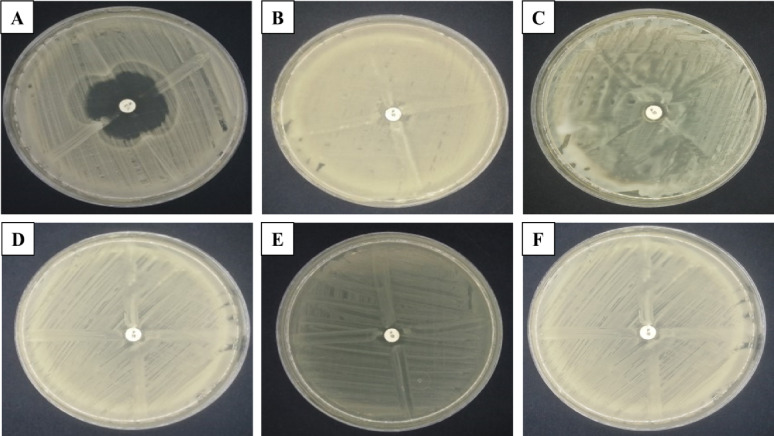
Detection of β-lactamase producing bacteria using cloverleaf test for *B. licheniformis*; **A**, *B. cereus* HES3; **B**, MRSA; **C**, *E. coli* D8; **D**, *P. aeruginosa*; **E**, and *K. pneumoniae* H4; **F**

This method was used as a simple, affordable, and reliable way to identify β-lactamase producing bacteria. *B. licheniformis* exhibited a distinct cloverleaf appearance when compared to *B. cereus* HES3, and MRSA (Fig. 2). However, *E*. *coli* D8, *P. aeruginosa*, and *K. pneumoniae* H4 showed a negative cloverleaf pattern.

### Masuda double-disc test

**Fig. 3:**
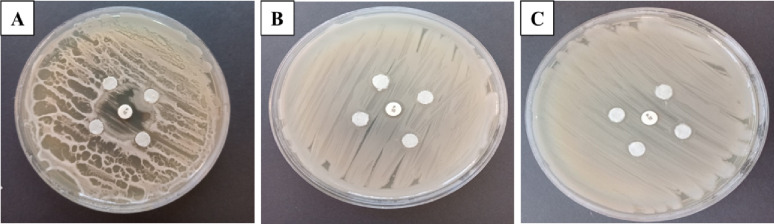
Masuda double-disc test for *B. licheniformis*; **A**, *B. cereus* HES3; **B**, and MRSA; **C**

In the Masuda double-disc test, a positive result indicating β-lactamase production was observed as inhibitory distortion around the centrally inserted disc. This was notably more pronounced and confirmed for *B. licheniformis* compared to *B. cereus* HES3, and MRSA (Fig. 3).

### ESBL test

**Fig. 4:**
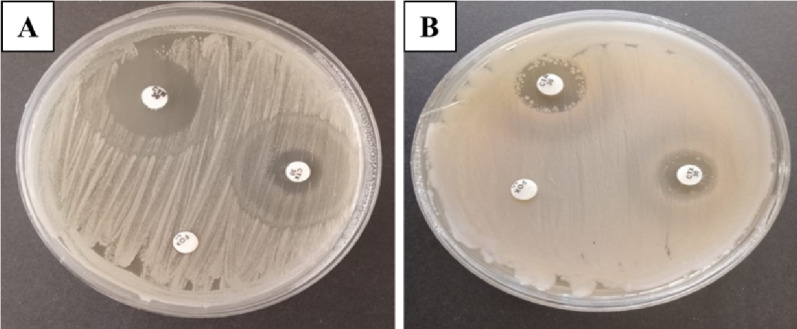
Extended-spectrum beta-lactamase test for *B. licheniformis*; **A**, and *B. cereus* HES3

*Bacillus licheniformis* and *B. cereus* strains produced extended spectrum beta-lactamase (ESBL) enzymes, specifically classified as class C enzymes (AmpC). The inhibition zone diameters were measured at 16 ± 0.3 mm for FOX, 15 ± 0.2 mm for CTX, and 18 ± 0.3 mm for CTR (Fig. 4). The sensitivity results of the Vitek 2 system for detecting ESBLs revealed that a *B. licheniformis* strain that had acquired penicillinases was classified as an ESBL using the composite reference standard.

### Biosynthesis of ZnO NPs

**Fig. 5:**
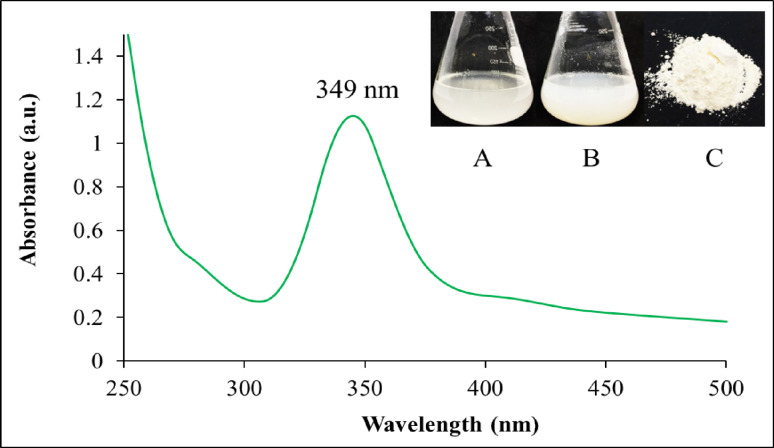
The UV-Vis spectrum of ZnO NPs. **A** The color of the reaction mixture at the beginning of the biosynthesis of ZnO NPs. **B** The color change into white colloidal precipitate after the production of ZnO NPs. **C** Yellowish white powder of ZnO NPs

The intracellular synthesis of ZnO NPs by *B. licheniformis* was indicated by color change to a white colloidal precipitate (Fig. 5). The synthesis of ZnO NPs was further confirmed by UV-Vis spectroscopy analysis, which showed a prominent peak at a wavelength of 349 nm. After the extraction, washing, and drying processes, a yellowish-white powder of ZnO NPs was obtained (Fig. 5).

### Characterization of ZnO NPs and ZnO/CS/VA

**Fig. 6:**
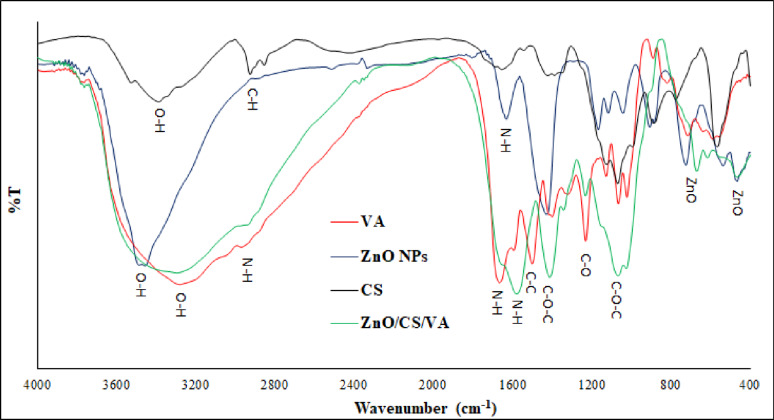
FTIR spectrum of VA, ZnO NPs, CS, and ZnO/CS/VA

The FTIR spectra of the prepared samples were measured in the range of 4000–400 cm^-1^ and are presented in Fig. 6. The detected peaks were 3258, 2941, 1665, 1553, 1448, 1335, 1075 and 549 cm^-1^. The peak observed at 3258 cm^-1^ indicates the presence of stretching and bending vibrations caused by hydroxyl groups. N − H bends in primary amines typically have a peak between 1553 and 1665 cm^-1^, while asymmetric amines have a peak at 2941 cm^-1^. Stretching bands for C − O esters, alcohols, ethers, phenols, and carboxylic acids were recorded in the range of 1075–1335 cm^-1^. The peak for C − C stretch (in-ring) aromatics was at 1448 cm^-1^. The vibration stretching bands of ZnO were detected in both the spectra of ZnO NPs and ZnO/CS/VA at 461–720 cm^-1^ confirming the successful loading of ZnO with the prepared nanocomposite.

**Fig. 7:**
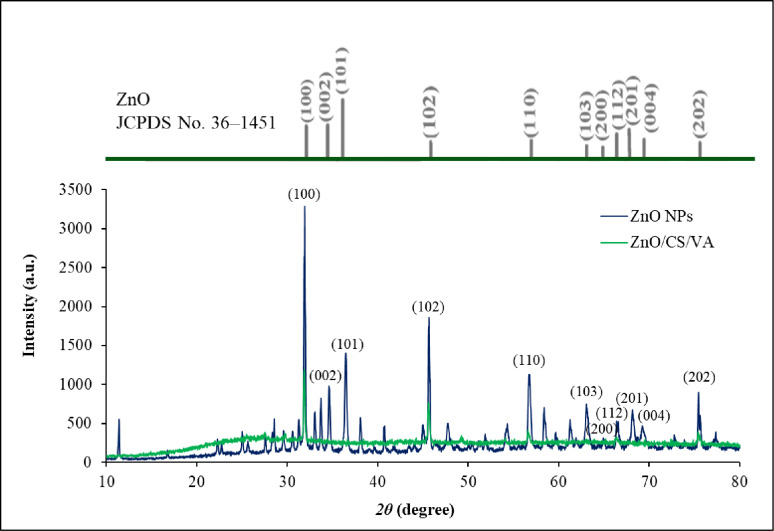
XRD patterns of ZnO NPs and ZnO/CS/VA

A typical XRD pattern of the synthesized ZnO NPs is shown in Fig. 7. Sharp diffraction peaks are observed, indicating the formation of well-crystalline materials. The spectrum of ZnO NPs displayed peaks appearing at 31.97°, 34.63°, 36.45°, 47.72°, 56.68°, 61.92°, 66.40°, 67.62°, 69.25°, 73.22°, and 75.44° corresponding to the lattice planes of ZnO; (100), (002), (101), (102), (110), (103), (200), (112), (201), (004) and (202), respectively. All characteristic peaks observed for ZnO NPs are in good agreement with those listed in the Joint Committee of Powder Diffraction Standards (JCPDS) card No. 00-036-1451, with a = 0.3249 nm, c = 0.5206 nm (Table 2) [93]. The ZnO NPs integrated within ZnO/CS/VA were analyzed to determine their typical crystal size and composition using XRD analysis at the lattice planes (100), (102), (110), (200), and (202) (Table 2). According to the spectrum of ZnO/CS/VA, the peaks appearing at 31.88°, 47.58°, 56.62°, 66.35°, and 75.41° correspond to the lattice planes of ZnO; (100), (102), (110), (200), and (202), respectively. These peaks are characteristic of the Wurtzite hexagonal crystal structure. The Scherrer equation (*D = kλ/ βcosθ*) was utilized to calculate the size of particles, where *D* represents particle size, *K* is a constant equal to 0.94, *λ* stands for x-ray wavelength, *β* represents the full width at half maximum, and *θ* indicates the angle of diffraction. The average particle size was determined to be 79.38 nm.

**Table 2:** Comparison between measurement XRD results and standard pattern for pure ZnO

hkl	Standard 2θ (°)	Observed 2θ (°)	
		ZnO NPs	ZnO/CS/VA
100	31.769	31.966	31.878
002	34.421	34.628	-
101	36.252	36.446	-
102	47.538	47.715	47.577
110	56.602	56.678	56.621
103	62.862	61.916	-
200	66.378	66.399	66.353
112	67.961	67.621	-
201	69.100	69.248	-
004	73.466	73.224	-
202	75.478	75.443	75.417

**Fig. 8:**
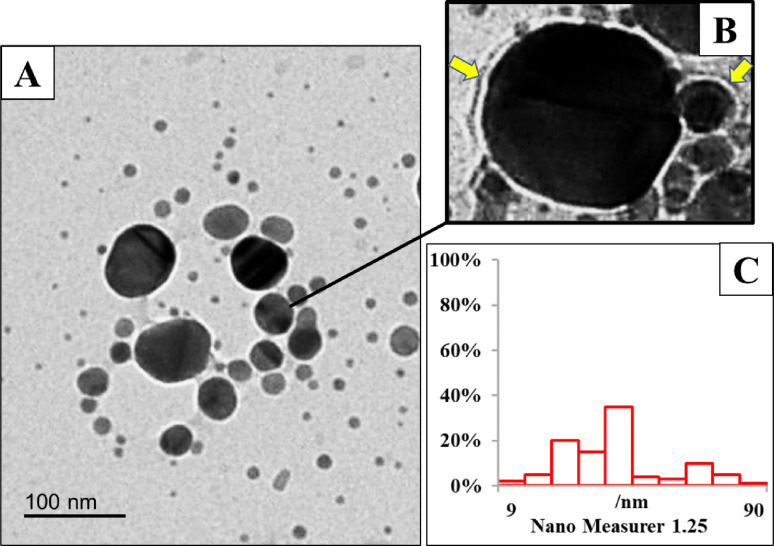
**A** TEM micrograph of ZnO/CS/VA with scale bar = 100 nm. **B** Magnified TEM micrograph of a core of ZnO NPs surrounded by a coat of a mixture of CS and VA. Yellow arrow denotes ZnO NPs coated by CS/VA shell. **C** Nanogravimetric image showing the particle size distribution of ZnO/CS/VA

As shown in Fig. 8, TEM confirmed the successful synthesis of ZnO/CS/VA. The ZnO NPs had a spherical appearance with an average size ranging from 33.24 to 69.11 nm, consistent with the XRD results. This green nanocomposite is composed of a nano-core of biosynthesized ZnO NPs, which is surrounded by a degradable CS coating containing VA as a drug (Fig. 8B). The nano-measurement graph shows a maximum size of 81 nm (frequency = 5%) and a minimum size of 18 nm (frequency = 5%) (Fig. 8C).

**Fig. 9:**
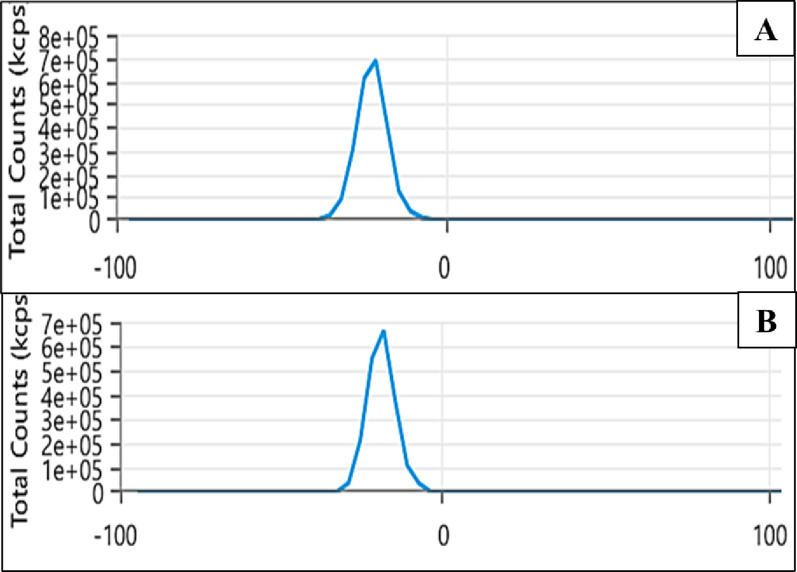
Zeta potential of ZnO NPs; **A**, and ZnO/CS/VA; **B**

The zeta potential results showed the negative charge of the biosynthesized ZnO NPs (-22.54 mV) and the green prepared nanocomposite (-17.78 mV) as shown in Fig. 9.

### Antibacterial activity

**Fig. 10:**
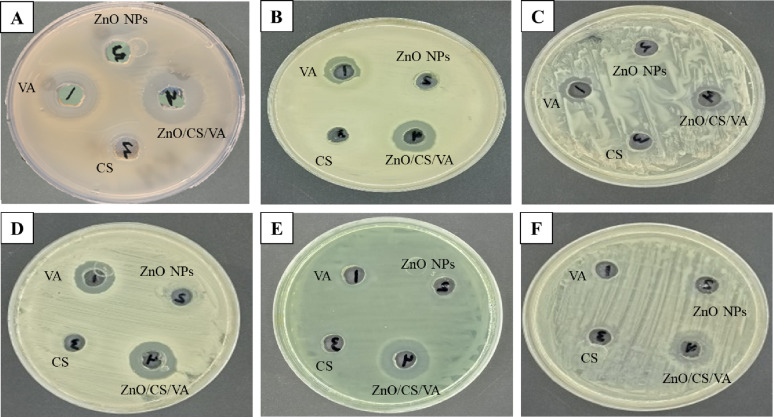
Antibacterial activity of VA, ZnO NPs, CS, and ZnO/CS/VA against *B. licheniformis*; **A**, *B. cereus* HES3; **B**, MRSA; **C**, *E. coli* D8; **D**, *P. aeruginosa*; **E**, and *K. pneumoniae* H4; **F**

The agar well diffusion test showed that ZnO/CS/VA exhibited good antibacterial activity against *B. licheniformis* (23.9 ± 0.19 mm), followed by *P. aeruginosa* (19.7 ± 0.03 mm), *E. coli* D8 (17.6 ± 0.03 mm), *K. pneumoniae* H4 (16.5 ± 0.21 mm), *B. cereus* HES3 (14.8 ± 0.06 mm), and MRSA (13.1 ± 0.18 mm), respectively (Fig. 10; Table 3). It was also noted that bacterial strains including *B. cereus* HES3, MRSA, *E. coli* D8, and *P. aeruginosa* showed resistance to CS. Additionally, MRSA and *K. pneumoniae* H4 showed complete resistance to the biosynthesized ZnO NPs. ZnO/CS/VA exhibited superior antimicrobial activity compared to the standard antimicrobial VA. Overall, ZnO/CS/VA showed a stronger bactericidal effect against the tested Gram-negative bacteria than the Gram-positive bacteria with the exception of *B. licheniformis*.

The visual data (Fig. 10) indicates that the ZnO/CS/VA nanocomposite exhibits the largest zone of inhibition against nearly all tested strains. The ANOVA followed by a Tukey’s post-hoc test (*p* < 0.05) was performed to compare the zone of inhibition of the composite against the individual components (VA, ZnO NPs, and CS). The statistical analysis formally confirmed the superior efficacy of the ZnO/CS/VA nanocomposite. The ANOVA demonstrated a significant effect of the antimicrobial agent on the zone of inhibition for every strain tested (*p* < 0.05 for all comparisons). Specifically, the post-hoc analysis (Table 3) revealed that the ZnO/CS/VA nanocomposite exhibited a statistically significant larger zone of inhibition (denoted by the unique superscript ‘a’) compared to all individual components against *B. cereus* HES3, *E. coli* D8, *P. aeruginosa*, and *K. pneumoniae* H4. Against *P. aeruginosa*, the ZnO/CS/VA zone (19.7 mm) was more than 2.8-fold larger than the most active individual component (VA, 6.8 mm), strongly aligning with the synergistic effect hypothesis between the nanocomposite parts. Against MRSA and *B. licheniformis*, the ZnO/CS/VA nanocomposite was statistically equivalent to the most active single component (VA), as indicated by the shared superscript ‘a’. However, even in these cases, the composite demonstrated the numerically largest zone and was statistically superior to the other two components (ZnO NPs and CS). These results move beyond visual evidence, providing formal statistical proof that the ZnO/CS/VA nanocomposite design is highly effective at enhancing antibacterial activity, particularly against difficult Gram-negative strains.

**Table 3:** Agar well diffusion method of VA, ZnO NPs, CS, and ZnO/CS/VA against the tested bacterial strains

Antimicrobial agents	Zone of inhibition (mm, mean ± SD)					
	Gram-positive bacteria			Gram-negative bacteria		
	B. licheniformis	B. cereus HES3	MRSA	E. coli D8	*P*. aeruginosa	K. pneumoniae H4
VA	22.3 ± 0.14^ab*^	11.9 ± 0.21^b^	12.8 ± 0.23^a^	15.6 ± 0.03^b^	6.8 ± 0.18^b^	-ve^c^
ZnO NPs	20 ± 0.03^b^	8.7 ± 0.16^c^	-ve^b^	6.0 ± 0.16^c^	6 ± 0.21^b^	-ve^c^
CS	12.5 ± 0.06^c^	-ve^d^	-ve^b^	-ve^d^	-ve^c^	10.8 ± 0.24^b^
ZnO/CS/VA	23.9 ± 0.19^a^	14.8 ± 0.06^a^	13.1 ± 0.18^a^	17.6 ± 0.03^a^	19.7 ± 0.03^a^	16.5 ± 0.21^a^

^*^Data represents mean ± SD (*n* = 3). Means followed by the same superscript letter within the same column are not statistically different (*p* > 0.05) according to the Tukey’s post-hoc test. -ve denotes no zone of inhibition.

**Fig. 11:**
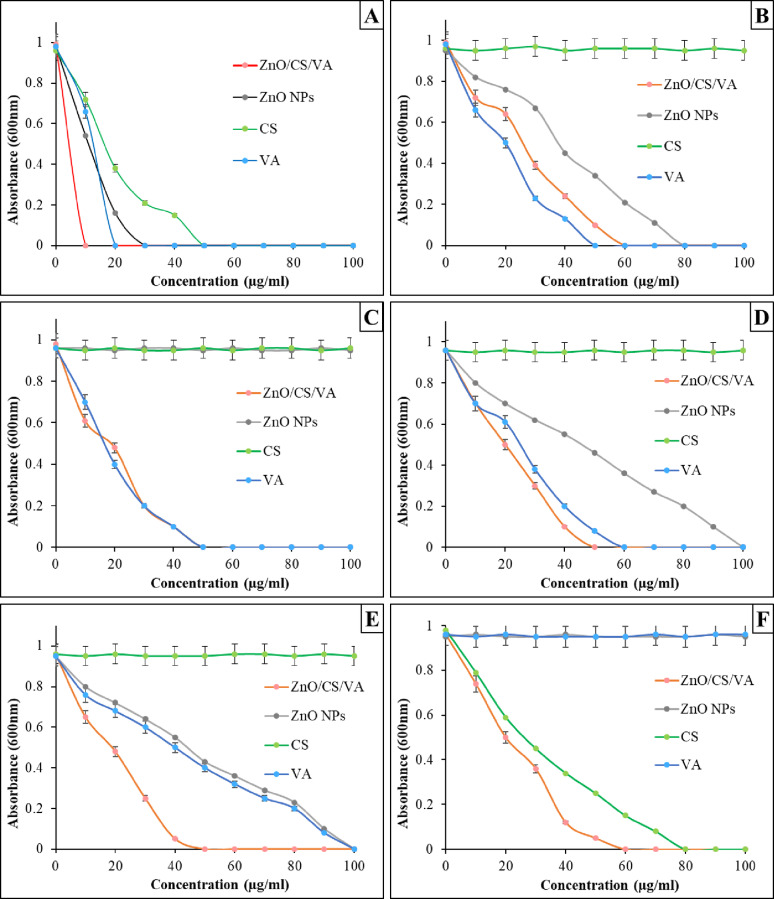
MIC test of VA, ZnO NPs, CS, and ZnO/CS/VA against *B. licheniformis*; **A**, *B. cereus* HES3; **B**, MRSA; **C**, *E. coli* D8; **D**, *P. aeruginosa*; **E**, and *K. pneumoniae* H4; **F**

**Fig. 12:**
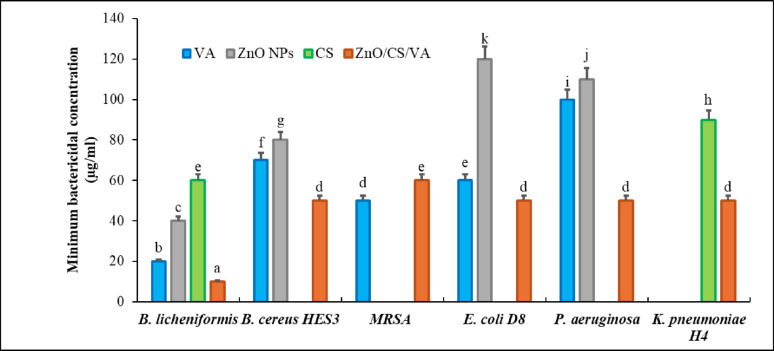
MBC test of VA, ZnO NPs, CS, and ZnO/CS/VA against the tested bacterial strains

MIC and MBC investigations were also conducted for ZnO NPs and ZnO/CS/VA in comparison to CS and VA (Figs. 11 and 12). In the MIC test, certain MHB cultures containing CS, VA, ZnO NPs, and ZnO/CS/VA exhibited bacterial turbidity after 24 h of incubation at 37 °C, indicating bacterial growth. Conversely, no turbidity was observed at other concentrations, suggesting bacterial growth inhibition (MIC values). In the current study, the MIC values of ZnO NPs were 30 µg/mL against *B. licheniformis*, 80 µg/mL against *B. cereus* HES3 and 100 µg/mL against *P. aeruginosa* and *E. coli* D8. Conversely, ZnO/CS/VA exhibited the most effective MICs against all bacterial strains tested. All antibacterial agents tested demonstrated an inhibitory effect that was dose-dependent. It was confirmed to be bactericidal when the suspension from MHB cultures supplemented with 60 µg/ml of ZnO/CS/VA was inoculated on MHA plates and incubated for 24 h. No bacterial growth was observed in any of the tested bacterial strains. The concentrations of ZnO/CS/VA mentioned are required to completely inhibit both Gram-positive and Gram-negative bacteria, indicating its broad-spectrum action. When the concentration of ZnO NPs was between 100 and 120 µg/ml, VA was necessary to induce a bactericidal effect against *B. cereus*, *E. coli* D8, and *P. aeruginosa*. It was interesting to note that the MBCs of ZnO/CS/VA against the tested bacterial strains were consistent with their MICs confirming its strong bactericidal properties compared to other substances. VA exhibited lower MBC values than ZnO NPs.

In order to formally validate the rationale for combining ZnO, CS, and VA, and to confirm that the enhanced activity of the ZnO/CS/VA composite is truly synergistic and not merely additive, the fractional inhibitory concentration (FIC) index was determined for the tested bacterial strains (Table 4). Since the FIC index is commonly used for binary systems, we calculated the FIC_Ratio_ by comparing the MIC of the full composite (ZnO/CS/VA) to the MIC of the most active single component (VA or ZnO NPs or CS) for each strain [94, 95]. The quantitative FIC analysis provides crucial insight into the interaction between the components of the composite. A synergistic activity (FIC_Ratio_ = 0.50) against the bacterial strains; *B. licheniformis* and *P. aeruginosa* was recorded. The synergy against the Gram-negative *P. aeruginosa* is particularly significant. It demonstrates that the strategic combination of ZnO and CS effectively overcomes the challenging outer membrane permeability barrier, allowing the components to act collaboratively [96, 97]. Against MRSA, the FIC_Ratio_ of 1.00 confirms a purely additive effect. This means that the composite is just as effective as the most active single component (VA), but it can potentially be delivered more efficiently through the nanocarrier system [98]. Additive effects were also observed for *E. coli* D8 (0.83) and *K. pneumoniae* H4 (0.75). However, the composite demonstrated antagonism (FIC_Ratio_ = 3.00) against *B. cereus* HES3. This unexpected finding warrants further investigation, but suggests that the relative concentrations of the individual components within the ZnO/CS/VA composite, when tested against this specific strain, may interfere with optimal activity. Despite this, the confirmed synergy against *P. aeruginosa* and *B. licheniformis* validates our core hypothesis that the strategic multi-modal combination can yield superior performance against selected pathogens.

**Table 4:** Fractional inhibitory concentration index analysis demonstrating synergistic and additive effects of the ZnO/CS/VA nanocomposite against the tested bacterial strains

Bacterial strain	MIC_Individual_ (µg/ml) (Most Active)	MIC_composite_ (µg/ml)	FIC_Ratio_ (Composite/Individual)	Interpretation (FIC ≤ 0.5 is synergy)
*B. licheniformis*	20 (VA)	10	0.50	Synergistic
*B. cereus* HES3	20 (VA)	60	3.00	Antagonistic
MRSA	50 (VA)	50	1.00	Additive
*E. coli* D8	60 (VA)	50	0.83	Additive
*P. aeruginosa*	100 (ZnO NPs and VA)	50	0.50	Synergistic
*K. pneumoniae* H4	80 (CS)	60	0.75	Additive

### CT-DNA binding investigation

**Fig. 13:**
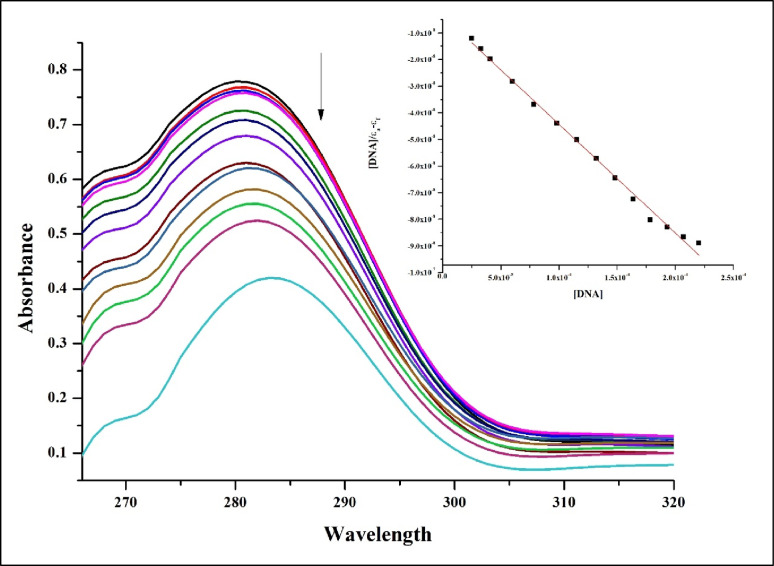
ZnO/CS/VA absorption spectrum in a pH 7.2 buffer with the increasing CT-DNA concentration. The arrow illustrates how absorbance varies as CT-DNA concentration rises

Using electronic absorption spectroscopy, we quantified and identified the intercalation of ZnO/CS/VA with CT-DNA (Fig. 13). The experiments with CT-DNA were conducted at room temperature using specific concentrations of ZnO/CS/VA. The concentration of CT-DNA was gradually increased, and the absorption spectra were measured to determine the intrinsic binding constant. The results showed that the absorption spectra decreased with increasing CT-DNA concentrations of ZnO/CS/VA, exhibiting a slight hypochromism effect. The predicted K_b_ value for ZnO/CS/VA was determined to be 1.17 × 10^5^ M^− 1^ using the absorption spectrum method. Hypochromism was observed when ZnO/CS/VA intercalated with calf thymus DNA.

### Docking studies

Vancomycin demonstrated a stronger affinity compared to ZnO NPs (Table 5), with lower scores (-10.85 kcal/mol vs. -2.73 kcal/mol for 3T88; -9.38 kcal/mol vs. -1.20 kcal/mol for 4DKI). The relatively higher RMSD values of VA (2.4–3.0 Å) may suggest conformational flexibility that allows for the accommodation of variable receptor geometries. However, this should be viewed as potential structural adaptability rather than definitive evidence of induced fit. These results are in line with the recommended docking interpretations by Morris et al. [99]. and Forli et al. [100].

**Table 5:** Docking scores and energies of ZnO NPs and VA with *E. coli* (PDB code: 3T88) and MRSA (PDB code: 4DKI)

Mol	Receptor	S	rmsd_refine	E_conf	E_place	E_score1	E_refine	E_score2
ZnO NPs	3T88	-2.73125	0.93436003	-1118.71	-41.9501	-2.74677	6.808312	-2.73125
VA	3T88	-10.8546	2.4072533	-158.3559	-16.2251	-0.74429	-52.001	-10.8546
ZnO NPs	4DKI	-1.19506	1.7127199	-1119.85	-33.0493	-8.43408	1.087366	-1.19506
VA	4DKI	-9.38016	3.0425267	-160.2708	-36.1534	-7.241301	-50.381	-9.38016

The interactions are dispalyed at Table 6; Figs. 14, 15, 16, 17, and supplementary Figs. S1-S8 that occurred as follow:

- ZnO NPs: Metal coordination (Zn-O at 2.02 Å, -2.1 kcal/mol) and ionic bonds (O-GLU at 3.91 Å, -0.7 kcal/mol) dominated.
- VA: Hydrogen bonds (N-PHE: 3.17 Å, -1.6 kcal/mol; O-GLU: 2.93 Å, -6.0 kcal/mol) and H-pi interactions (C-TRP: 3.87 Å, -0.8 kcal/mol) were critical.
- RMSD: ZnO NPs exhibited lower RMSD values (0.93Å for 3T88; 1.71Å for 4DKI), indicating stable binding conformations compared to VA (2.41Å–3.04Å).

**Table 6:** Interaction of ZnO NPs and VA with *E. coli* (PDB code: 3T88) and MRSA (PDB code: 4DKI)

Bacteria	Ligand	Atom Involved	Binding residues	Interaction Types	Distance (Å)	Binding Energy (kcal/mol)
*E. coli* (PDB code: 3T88)	ZnO NPs	O 3	N PHE 162 (A)	H-acceptor	2.88	-0.7
		Zn 2	O PHE 162 (A)	Metal	2.02	-2.1
	VA	O 156	N PHE 162 (A)	H-acceptor	3.17	-1.6
		N 172	OH TYR 94 (A)	H-acceptor	3.19	-0.7
		C 85	6-ring TRP 166 (A)	H-pi	3.87	-0.8
MRSA (PDB code: 4DKI)	ZnO NPs	ZN 10	O GLU 189 (A)	Metal	2.36	-1.5
		O 1	OE2 GLU 379 (A)	Ionic	3.91	-0.7
	VA	C 92	OE1 GLU 222 (A)	H-donor	3.49	-0.5
		O 121	OE1 GLU 222 (A)	H-donor	2.93	-6.0
		O 16	NZ LYS 188 (A)	H-acceptor	3.15	-0.8

**Fig. 14:**
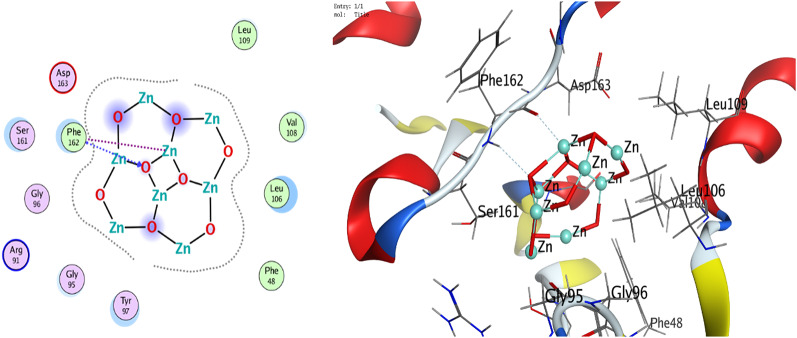
2D and 3D diagrams show the interaction between ZnO NPs and active sites of 3T88

**Fig. 15:**
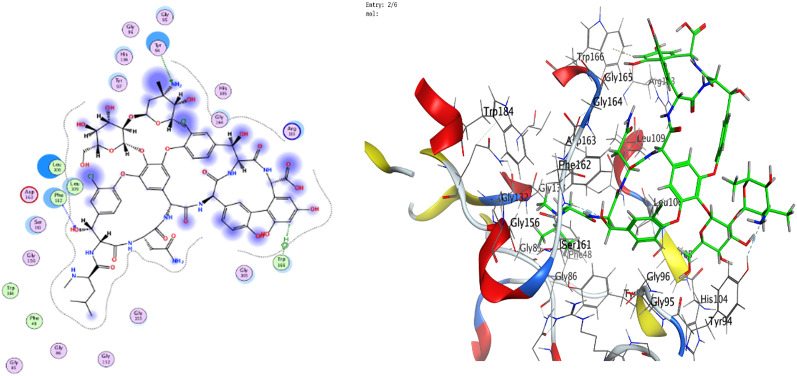
2D and 3D diagrams show the interaction between VA and active sites of 3T88 protein

**Fig. 16:**
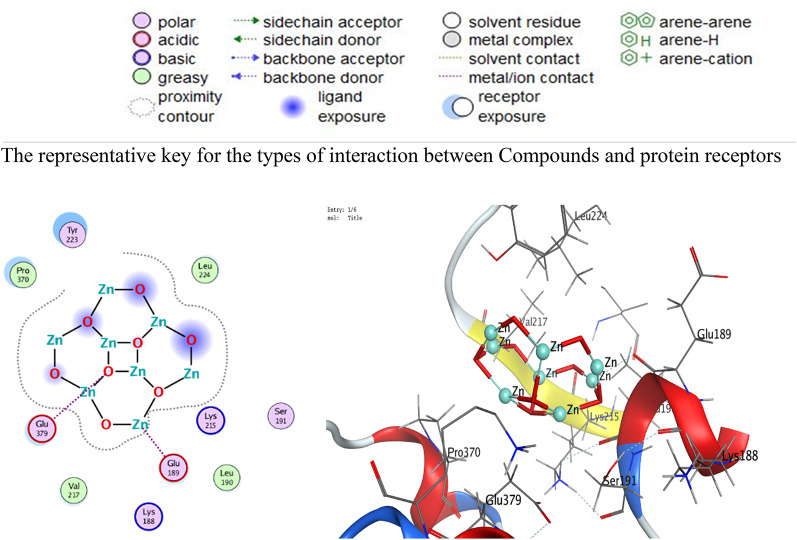
2D and 3D diagrams show the interaction between ZnO NPs and active sites of 4DKI protein

**Fig. 17:**
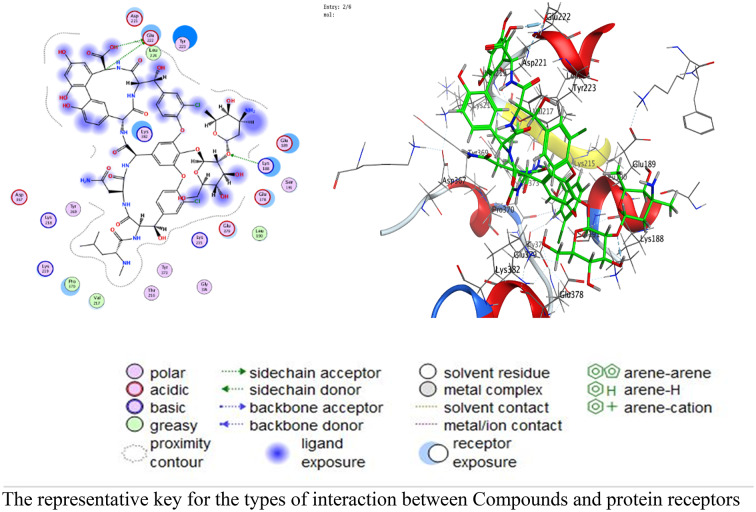
2D and 3D diagrams show the interaction between VA and active sites of 4DKI protein

#### Investigation of cytotoxicity effect of ZnO NPs and ZnO/CS/VA

**Fig. 18:**
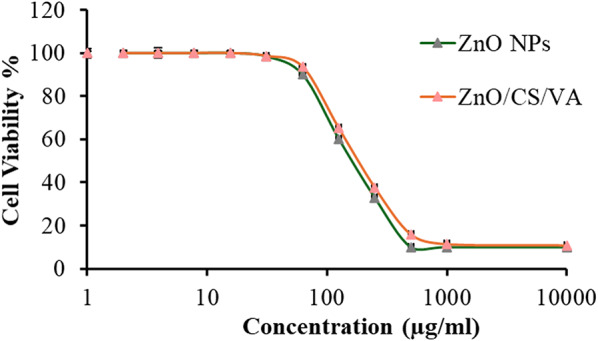
Cytotoxicity of ZnO NPs and ZnO/CS/VA aginst Vero cells

The toxicity of the biogenic ZnO NPs and green-prepared ZnO/CS/VA was tested and assessed using the Vero cell line (Fig. 18). ZnO NPs and ZnO/CS/VA exhibited remarkable and high CC_50_ values of 146.62 ± 1.03 and 162.86 ± 1.07 µg/mL, respectively. This suggests that both ZnONPs and their combination were not toxic to Vero cell lines. This result demonstrates that using green-prepared ZnO NPs and ZnO/CS/VA at high concentrations, such as MICs and MBCs (60–120 µg/mL) is safe.

## Discussion

Bacterial infectious diseases are a persistent health danger and one of the leading causes of death globally [101]. It is impossible to accurately assess the global cost of infectious diseases today due to their multifaceted impact on health, the economy, and other aspects of society. One of the biggest threats to global public health is MDR pathogens [102]. The current study found high resistance percentages among various Gram-positive and Gram-negative bacteria indicating that *B. licheniformis*, *B. cereus* HES3, MRSA, *E. coli* D8, *P. aeruginosa*, and *K. pneumoniae* H4 are resistant to different classes of antibiotics. Specifically, resistance rates reached 83.33% against ampicillin, 66.67% against amoxycillin/clavulanate, ceftriaxone, and cotrimoxazole, and 50% against cefepime, ciprofloxacin, erythromycin, and vancomycin. The results of Moş et al. [103]. revealed that 52.3% of clinical *E. coli* isolates were sensitive to imipenem and 38.6% to gentamicin, compared to the international statistic of 70%. However, the highest level of resistance was found for ampicillin (6.8%). This suggests that the isolated strains of *E. coli* are especially susceptible to carbapenem and third-generation aminoglycosides. Kibret & Abera [72] reported high prevalence of Enterobacteriaceae resistance to erythromycin (89.4%), amoxicillin (86.0%), and tetracycline (72.6%). According to Pokharel et al. [105]. , *P. aeruginosa* was primarily isolated from wound swabs and pus showing resistance to ceftazidime in 63.04% of cases, cefixime in 65.21%, ceftriaxone and cefotaxime in 56.52% of cases, and piperacillin in 56.52% of cases. Worku et al. [106]. recorded that 83.3% and 84% of *K. pneumoniae* clinical isolates exhibited resistance to tetracycline and ceftriaxone, respectively. Munawer et al. [107]. noticed high resistance of MRSA clinical isolates to penicillin, cefoxitin, and sulfamethoxazole.

Therefore, novel antibacterial agents are needed to effectively combat such microbes. Nanomaterials and nanodrug therapy are promising solutions to address bacterial resistance. Metallic and metallic oxide NPs can also affect the stability, efficacy, and release of drugs [108]. These nanoparticles provide a new therapeutic treatment for bacterial infections and drug-resistant strains [29, 83, 109]. NPs activities can target multiple biomolecules, potentially showing or halting the emergence of many MDR pathogens [110, 111].

Drug delivery-specific NPs can encapsulate target drugs within the particles, shielding them from enzymatic breakdown in the biological system [112, 113]. Because of their subcellular size, NPs can assist in the absorption of drugs more readily within cells. These results in a lower concentration of free pharmaceuticals reducing their harmful effects [114]. ZnO nanomaterials offer special semiconductor and piezoelectric qualities in addition to being biocompatible and biodegradable [115]. ZnO nanocomposites have been used in manufacturing due to their excellent mechanical, anti-ultraviolet, photocatalytic, thermal stability, hydrophobicity, as well as multifunctional antibacterial film and antibacterial paper properties [116]. Experiments have demonstrated that these materials have excellent mechanical, anti-ultraviolet, and antibacterial properties [117]. In this investigation, the addition of aqueous Zn^2+^ to cell-biomass-derived *B. licheniformis* resulted in the reduction of Zn ions into ZnO NPs [81]. Markus et al. [118]. suggested that biological substances and functional groups on the bacterial cell were responsible for the reduction of Zn^2+^ to ZnO NPs. This bacterium biosynthesized ZnO NPs over 24-hour period, which was consistent with Hussein et al. [119]. , who demonstrated how *B. cereus* can be used as a bio-templating agent to produce ZnO NPs. Ali et al. [120]. utilized *B. subtilis* to create a straightforward and cost-effective method for biosynthesizing of ZnO NPs. Furthermore, *Aeromonas hydrophila* successfully synthesized ZnO NPs within a 24-hour timeframe at room temperature [121]. ZnO NPs were produced using *Lactobacillus* sp. over a period of 12 h. This was achieved by heating the reaction mixture to 80 °C for 5–10 min on a water bath [122].

The observation of color changes in the reaction suspension served as the first screening method for the synthesis mediated by cell biomass [81]. The bacterial cell biomass appeared as clear white in distilled water. The color of the solution changed to cloudy white after the addition of Zn^2+^ indicating that Zn^2+^ was reduced to ZnO NPs. UV–Vis spectroscopy was utilized to monitor the reduction of Zn^2+^ to ZnO NPs within the range of 300 to 700 nm. The UV-Vis spectroscopy analysis was utilized as additional screening method for the formation of ZnO NPs, revealing an absorbance peak at 349 nm in the recovered ZnO NPs. This range of absorption peaks has also been observed in previous studies on the production of ZnO NPs using various microorganisms [123, 124]. This UV absorption peak appears to be similar to the absorption band identified in the findings of Hudlikar et al. [125]. who discovered that the absorption peaks of ZnO NPs fall between 340 and 385 nm. In addition, the absorption peak obtained also matches with the ZnO NPs derived from the cell-free supernatant and the cell biomass of *Lactobacillus plantarum* TA4, which had deep peaks at 349 and 351 nm, respectively [81].

ZnO NPs have been documented as effective antibacterial agents. However, Zhang et al. [126]. reported that, it was possible for bacteria to develop resistance and grow at high ZnO concentrations (1000 µg/mL) during their assessment of how ZnO NPs affected *E. coli*. The antibacterial sensitivity returned after the NPs were removed from the growing media for a few days. This temporary resistance is adaptive and primarily results from bacterial shape changes rather than genetic alterations.

When exposed to stress, the oval-shaped bacteria that are resistant changed to the more common rod-like form [127]. As a result, several studies are focused on developing and enhancing the antibacterial properties of ZnO NPs against resistant strains. Many biopolymers such as chitosan (CS), cellulose, gelatin, and others, have been incorporated with NPs to develop therapeutic agents. However, combinations with CS have been reported as the safest and most effective antimicrobial agents [58]. CS is a biopolymer used to create metallic NPs by serving as a reducing and protecting agent [128]. Furthermore, the polymeric material has a high rate of biodegradability and biocompatibility due to its natural and organic nature. There is interest in employing CS because of its well-founded cationic and polymeric characteristics. Additionally, its accessibility, non-toxicity, and antibacterial properties, make it a very intriguing option for use as biomaterial in applications involving medicine, food, and industry. CS and CS NPs have been extensively researched for their potential usage in the field of biology due to their small size and high surface to weight ratio. Therefore, the current study aimed to combine VA with CS and ZnO NPs to create a new drug delivery system. The study also sought to investigate the antibacterial potential and DNA binding ability of this system against some MDR bacteria.

The protonation of CS’s amino groups causes it to dissolve in acetic acid, making it soluble in water and producing a viscous solution that is ideal for film formation. To stabilize the NPs within the CS matrix and prevent aggregation, ZnO NPs interact with CS through electrostatic interactions, hydrogen bonds, and van der Waals forces [129]. FTIR measurements were used to identify the potential biomolecules that effectively stabilized and capped the metal NPs [130]. However, the FTIR data was used to identify the probable biomolecules responsible for effectively stabilizing and capping the ZnO NPs produced by *B. licheniformis*. The results indicated that FTIR analysis confirmed the presence of carbonyl groups from amino acid residues, showing that proteins have a strong affinity for binding metal from metal NPs. This ability to cap ZnO NPs residues could potentially inhibit the accumulation and aggregation of NPs. This suggests that biological molecules can both form and remove metal NPs [131, 132]. The vibration stretch of ZnO NPs has been determined at 549 cm^− 1^. In other studies, FTIR spectra of ZnO NPs showed Zn − O stretches at 466.77 cm^− 1^, 482 cm^− 1^, 513 cm^− 1^, 515 cm^− 1^, 584 cm^− 1^ and 612 cm^− 1^ [56, 133–136]. The FTIR spectra provide critical evidence for the successful formation and chemical interaction within the ZnO/CS/VA nanocomposite. The ZnO NPs was observed aligning on the VA and CS matrix, suggesting successful integration. Evidence of their interaction with the ZnO NPs was shown by a decrease in the strength of the vibration bands of the − OH, −NH, and C–O groups in the ZnO/CS/VA FTIR spectrum. The broad, intense peak corresponding to the O-H stretching vibrations (hydrogen bonding) in CS at 3400 cm^− 1^ shows a notable shift and decreased intensity in the ZnO/CS/VA composite. This is accompanied by shifts in the C-O-C peaks around 1080 cm^− 1^ and the N-H bending peak around 1590 cm^− 1^. These collective changes confirm that the hydroxyl and amino groups of CS are successfully involved in coordination and hydrogen bonding with the surface of the ZnO NPs [137]. The presence of characteristic ZnO peaks in the low fingerprint region (400–600 cm^− 1^) in the final composite further confirms the successful inclusion of the NPs [138]. The characteristic N-H stretching peak of VA at 3290 cm^− 1^ and the amide carbonyl bands (C = O stretch) at 1650 cm^− 1^ show significant shifts and broadening in the final composite spectrum. This indicates that the amino and peptide groups of VA are actively participating in hydrogen bonding or weak coordination with the CS matrix and the ZnO surface [17]. The resulting spectral changes confirm the effective loading and chemical binding of VA onto the ZnO/CS platform, validating the prepared nanocomposite structure.

Capping agents may contain either negative or positive charges, which are essential for maintaining the stability of NPs and ensuring their colloidal dispersion [139, 140]. The repulsion forces between NPs due to the presence of significant charges prevent agglomeration, ensuring the stability of the NPs [141]. The stability of the NPs was demonstrated by the magnitude of the zeta potential value. The prepared NPs have a negative charge on their surface, as shown by the zeta potential measurements. According to the findings, ZnO NPs had a negative zeta potential of -22.54 mV while ZnO/CS/VA had − 17.78 mV. Abdelhakim et al. [142]. , a negative zeta potential of ZnO NPs reached − 23.92 mV, indicating that the ZnO NPs were highly stable. Ebadi et al. [143]. used the cyanobacterium *Nostoc* sp. EA03 to biosynthesize ZnO NPs. The negative zeta potential value of -29.7 indicates that the bioactive compounds contribute to the long-term stability of the NPs.

The XRD patterns of the synthesized ZnO NPs showed that all of their diffraction peaks matched the conventional ZnO NPs data [144, 145]. Every characteristic peak found for ZnO NPs aligned well with those found on card No. 36-1451 of the JCPDS [146]. These findings demonstrate that the process used in this work to produce pure ZnO NPs is efficient. Furthermore, the results show that the diffraction peaks became smaller and more intense as the annealing temperature increase, suggesting that the ZnO NPs produced had an excellent crystalline structure. Additionally, the broadening at the base of the diffraction peaks in the XRD pattern indicates that the crystalline diameters of the ZnO NPs were modest and consistent with those reported in the literature [147]. Furthermore, the absence of additional impurity-related diffraction peaks confirms the high purity of the produced materials. Several studies suggest that the efficiency of the antibacterial activity of NPs is influenced by the size of the particles [148–150]. The observed attenuation and broadening of the characteristic ZnO peaks including the intense (100) and (101) reflections in the ZnO/CS/VA composite do not indicate a complete collapse of the ZnO crystal structure. Instead, this is mainly due to a masking effect combined with surface passivation. The thick, highly amorphous CS layer coating, along with the loaded VA, creates a significant amorphous background signal that effectively shields and reduces the diffraction intensity from the crystalline ZnO core [151]. Furthermore, the surface coordination between CS and the ZnO surface may induce a minor degree of surface lattice disorder, contributing to the broadening [152]. The remaining subtle peaks confirm that the ZnO component successfully retains its core Wurtzite hexagonal crystal structure within the final nanocomposite. The reduced intensity and broadening are indicative of successful polymeric encapsulation and surface passivation [153].

Numerous researches indicate that the size of the particles affects how efficient the antibacterial activity of NPs is [148–150]. According to Gold et al. [154]. , smaller particles have a stronger antibacterial effect than larger ones. They can harm microbial protoplasts by condensing DNA molecules, preventing replication, deactivating enzymes and metabolism, and impairing membrane permeability. The average size of ZnO/CS/VA was approximately ≈ 79.38 nm, which corresponded to the TEM micrograph. A visual contrast between the electron-dense ZnO core and the polymeric shell (CS/VA) provided improved evidence of the core-shell morphology. The image clearly depicts the core-shell structure, where the dark, electron-dense ZnO NPs forms the core, surrounded by a lighter, less electron-dense layer corresponding to the amorphous CS shell [155, 156]. This visualization confirms that the CS polymer successfully encapsulated the ZnO NPs, which is the physical basis for the stabilization and drug delivery mechanism proposed in the Introduction. Thus, coating process added extra thickness to the original metal NP core [157, 158]. Several studies on antibacterial materials such as Zn and Ag have shown that the antibacterial action is improved with an increase in surface-to-volume ratio. Additionally, microbial cell walls and cytoplasmic membranes may be negatively impacted by small particles [159]. A study by Kasemets et al. [160]. examined the antibacterial activity of ZnO particles at both the micro and nanoscales against *P. fluorescens*,* E. coli*, and *B. subtilis.* They concluded that ZnO NPs with an average size of 20 nm and a zeta potential of -5 mV were more effective against bacteria than microparticles. When compared to the controls, the NPs completely decreased the viability of all bacterial species, whereas the micro-size ZnO only slightly inhibited bacterial growth [161].

ZnO NPs were found to be more efficient against Gram-positive than Gram-negative bacteria while ZnO/CS/VA was effective against both Gram-positive and Gram-negative bacteria according to the antibacterial activity results. Since Gram-positive and Gram-negative bacteria have different cell wall compositions, it was discovered that ZnO NPs have greater antibacterial ability against the former [162]. On the plates, ZnO NPs and the concentration levels of 150 µg/mL significantly influenced the potential zone of inhibition against *B. licheniformis*, and *B. cereus* HES3 followed by *E. coli* D8 and *P. aeruginosa*.

According to the MIC and MBC results, high concentrations of ZnO NPs produced a complete inhibitory effect, as expected. On the other hand, low concentrations of ZnO/CS/VA act as a bactericidal agent with broad-spectrum action. The growth of bacteria was suppressed by ZnO/CS/VA at MIC of 10–60 µg/mL for *B. licheniformis*, *B. cereus* HES3, and MRSA, and 45–60 µg/mL for *E. coli* D8, *P. aeruginosa*, and *K. pneumoniae* H4. According to previous studies on the antibacterial properties of ZnO NPs and ZnO nanocomposites, the growth of *S. aureus*, MRSA, *E. coli*, *P. aeruginosa*, and *K. pneumoniae* was inhibited at MICs and MBCs ranging from 500 to 1500 µg/mL [56, 136, 163–165]. Higher concentrations of ZnO NPs and ZnO/CS/VA may suppress the growth of Gram-negative bacteria more effectively than Gram-positive bacteria, as indicated by the results of the MIC and MBC tests. The same findings were published by Emami-Karvani & Chehrazi [166], who emphasized that Gram-positive bacteria are more susceptible than Gram-negative bacteria. According to a study by Atmaca et al. [167]. , variations in cell wall construction, cell physiology, metabolism, or degree of contact may be linked to the increased vulnerability of Gram-positive bacteria.

Bhande et al. [168]. examined the synergistic effect of combining ZnO NPs with β-lactam antibiotics on spectrum β-lactamase producers commonly found in urinary tract infections. The ZnO NPs were combined with cefotaxime, ceftriaxone, ampicillin, and cefepime and tested on *E. coli*,* K. pneumoniae*,* S. paucimobilis*, and *P. aeruginosa*. The combination revealed enhanced bactericidal activity and induced membrane leakage, damaging the cell membrane, and ultimately leading to cell death. El-Telbany et al. [169]. reported that the combination of ZnO NPs and meropenem enhanced their antibacterial action and showed synergistic activity with lower MICs against multidrug-resistant *P. aeruginosa*. Aderibigbe [170] documented that the combination of ZnO NPs with tobramycin and gallic acid exhibited excellent antibacterial and antibiofilm activity against multidrug-resistant bacteria such as *P. aeruginosa*, *S. aureus*, and *E. coli*.

Although the exact mechanisms of ZnO’s antibacterial action are unknown, scientists have proposed a few potential bactericidal effects. Various NPs utilize different mechanisms to destroy bacteria. Metallic NPs use several modes of action to kill bacteria. They penetrate the bacterial cell wall and form pores on the surface of the membrane. This, in turn, causes the formation of free radicals that destroy the cell membrane [112, 171, 172]. On the other hand, Phan et al. [173]. reported that Zn ions can only prevent the growth of bacteria (bacteriostatic impact), not kill them (bactericidal effect). Due to the favored features of nanomedicines, multiple options for NPs have emerged including organic and inorganic nanosystems. The mechanism behind the combination of NPs and CS to enhance their antibacterial activity is most likely their interaction with the bacterial cell wall or membrane. Several theories have been proposed to explain this process. The most prominent is the CS NPs model of antibacterial activity, which focuses on the electrostatic interaction between the negatively charged bacterial cell membranes and the positively charged amino groups of glucosamine. The interaction brings about widespread surface alterations that change the membrane’s permeability. This leads to an osmotic imbalance and the release of internal components, ultimately causing cell death [174, 175].

Since DNA is a crucial part of the antibacterial process, the biological application of nanocomposites to investigate DNA interaction activity has attracted a lot of attention [176]. Using electronic absorption spectroscopy, the nanocomposite’s intercalation with calf thymus DNA was ascertained. The intrinsic binding constant (K_b_) of calf thymus DNA was tested. The tests were conducted at room temperature using fixed nanocomposite concentrations and a gradual increase in calf thymus DNA (CT-DNA) concentration. The absorption spectra of the nanocomposite showed a hypochromism effect as the CT-DNA content increased. This is because the intercalation method involves a strong stacking interaction between an aromatic chromophore and the base pairs of DNA, the intercalation of ZnO/CS/VA with calf thymus DNA demonstrates hypochromism. These findings provide strong evidence that substituting of a nanocomposite with a higher electron-donating ability for the terminal nitrogen facilitates the attachment of complexes to the DNA helix. The results of the electronic absorption tests clearly show that ZnO/CS/VA interact favorably with CT-DNA.

The comparative analysis of ZnO NPs and VA reveals nuanced insights into their binding mechanisms and therapeutic potential. VA shows superior docking scores against both *E. coli* (3T88) and MRSA (4DKI) receptors (-10.85 for 3T88; -9.38 for 4DKI) indicating its strong affinity. However, VA’s higher RMSD values (2.41–3.04 Å) suggest conformational flexibility during binding allowing it to adapt to diverse receptor geometries. In contrast, ZnO NPs exhibit a lower RMSD (0.93–1.71 Å), indicating rigid and stable interactions, likely a result of metal coordination (e.g., Zn-O at 2.02 Å) and ionic bonds. These interactions, while stabilizing their binding pose, restrict their ability to adapt to dynamic binding sites, therefore reducing efficacy against evolving pathogens. The strength of VA lies in its ability to form multiple high-affinity hydrogen bonds with critical residues. For instance, in 3T88, it forms an H-acceptor bond with N PHE 162 (3.17 Å, -1.6 kcal/mol) and a strong hydrogen bond with OH TYR 94 (-0.7 kcal/mol). In 4DKI, it interacts with OE1 GLU 222 through a strong hydrogen-bonding interaction (2.93 Å, -6.0 kcal/mol), which is a crucial residue for MRSA’s cell wall synthesis. These interactions appear to mimic natural substrate binding, enabling VA to competitively inhibit bacterial enzymes. In contrast, ZnO NPs depend on metal coordination (e.g., Zn-O at 2.02 Å) and weaker ionic bonds (-0.7 kcal/mol), which are stable but do not offer the same specificity as hydrogen bonds. PHE 162 and TYR 94 are located within a hydrophobic pocket in 3T88, crucial for substrate recognition in bacterial cell wall synthesis.

By binding here, VA competitively inhibits enzymatic activity. VA’s interactions with GLU 222 in 4DKI (MRSA) are particularly significant. This residue is part of the penicillin-binding protein (PBP) active site, which is a key target for β-lactam antibiotics. By forming a strong hydrogen bond here (-6.0 kcal/mol), VA disrupts cell wall synthesis, mirroring its actual mechanism of action. ZnO NPs’ metal coordination with GLU 189 (-1.5 kcal/mol) in 4DKI, while novel, targets a less critical region, explaining its weaker inhibitory potential. VA’s docking superiority aligns with its decades of clinical use against MRSA. Its hydrogen-bond-driven mechanism is less prone to interference from bacterial efflux pumps or enzymatic degradation compared to metallic NPs, which may face challenges in physiological environments (e.g., ion competition).

VA’s proficiency in hydrogen-bonding ensures high target specificity, minimizing off-target effects, which is crucial for systemic use. Additionally, its adaptability, characterized by flexible binding, as evidenced by higher RMSD values, allows for the accommodation of minor receptor conformational changes, thereby delaying resistance. VA also demonstrates synergy with other agents, making it suitable for use in combination therapies to enhance efficacy against resistant strains.

VA’s E_score2 values (-10.85 for 3T88; -9.38 for 4DKI) are significantly lower than the scores of ZnO NPs, indicating a stronger overall binding energy. This is further supported by its E refine term (-52.00 for 3T88; -50.38 for 4DKI), which demonstrates optimized pose refinement during docking. Non-biogenic NPs were prepared for several research projects and were discovered to be toxic substances with a high level of toxicity against specific normal cells potentially leading to genetic damage [177, 178].

However, studies have shown that naturally produced NPs are safe and environmentally friendly [54, 110, 179]. The toxicity of biogenic ZnO NPs and green-prepared ZnO/CS/VA was tested on the Vero cell line. Both ZnO NPs and ZnO/CS/VA showed high CC_50_ values of 146.62 ± 1.03 and 162.86 ± 1.07 µg/ml, respectively. This indicates that neither ZnONPs nor their combination were toxic to Vero cell lines.

This result demonstrates that using green-prepared ZnO NPs and ZnO/CS/VA at high concentrations, such as MBCs (60–120 µg/ml), is safe. The study reports that the cell-free extract of *B. licheniformis* ATCC 4527 has no apoptosis effect on the Vero cells at concentrations greater than 31.25 µg/mL of ZnONPs and its combination with CS and VA. Similarly, Sayed et al. [180]. found that concentrations lower than 31.25 µg/mL of nanocomposites may not exhibit cytotoxicity against Vero cells. In contrast, the cell-free extract of *B. megaterium* NCIM 2326 produced ZnO NPs with apoptotic effects at concentrations of ≥ 12.5 µg/ml, as reported by Saravanan et al. [181]. However, according to Yaghubi kalurazi & Jafari [182], when ZnO NPs were tested against Vero cells, they were able to inhibit the Vero cell lines.

According to Almuhayawi et al. [183]. , green synthesized ZnONPs also showed low toxicity on Vero cells, with a CC_50_ of 154.01 µg/mL. In comparison to the current results, Alrabayah et al. [184]. prepared green ZnO NPs that exhibit a lower cytotoxic CC_50_ of 145.77 µg/mL, but their combination displayed a higher CC_50_ reaching 179.23 µg/mL.

## Conclusions

A biosynthesis of ZnO NPs was prepared using *B. licheniformis* ATCC 4527. The NPs were then decorated with CS and VA to fabricate ZnO/CS/VA. Various physicochemical analyses were conducted on the prepared materials including UV-Vis spectroscopy, FTIR, Zeta potential, XRD, and TEM. High resolution TEM investigation is recommended for more study of the core/shell nanostructure of ZnO/CS/VA. The synthesized ZnO/CS/VA exhibited potent antibacterial activities in a dose-dependent manner. Results revealed that both Gram-positive and Gram-negative bacteria were sensitive to ZnO/CS/VA at low concentrations, with MIC values ranging from 10 to 60 µg/mL. The cytotoxicity, DNA binding, and docking studies of ZnO/CS/VA were also investigated confirming the strong antibacterial action of the prepared nanocomposite as well as its high safety profile. VA’s dominance in docking scores and interaction energy reaffirms its role as a potent antibiotic against both *E. coli* (3T88) and MRSA (4DKI) bacteria. Its hydrogen-bonding proficiency and adaptability provide a robust mechanism for targeting critical residues. Pairing VA with ZnO NPs could leverage both hydrogen bonding and metal-mediated interactions for dual-action therapy. This approach of enhancing traditional antibiotics may hold the key to addressing the antibiotic resistance crisis. Furthermore, future studies should demonstrate the in vivo efficacy of ZnO/CS/VA using an animal model.

## Supplementary Information

Below is the link to the electronic supplementary material.

Supplementary Material 1

## Data Availability

The datasets used and/or analyzed during the current study are available from the corresponding author on reasonable request.

## Abbreviations

AMR: Antimicrobial resistance
ESBL: Extended-spectrum beta-lactamases
ZnO NPs: Zinc oxide nanoparticles
ZnO/CS/VA: Zinc oxide/chitosan/vancomycin nanocomposite
TEM: Transmission electron microscopy
FTIR: Fourier transform infrared spectroscopy
XRD: X-ray diffraction
UV-Vis: Ultraviolet-visible spectroscopy
SD: Standard deviation
MIC: Minimum inhibition concentration
MBC: Minimum bactericidal concentration
CC_50_: Cytotoxic concentration 50%
MOE: Molecular operating environment
E_place: Score of the placement phase
E_conf: Energy conformer
E_refine: Score refinement
