# Supplementary material for: Antibacterial potential, DNA binding and molecular docking investigations of newly green synthesized zinc oxide/chitosan/vancomycin nanocomposite using Bacillus licheniformis ATCC 4527 against some drug-resistant bacteria
Source: Microb Cell Fact. 2026 Feb 11;25:56. doi: 10.1186/s12934-026-02928-9 (PMC12930810; doi:10.1186/s12934-026-02928-9)
Supplement: Supplementary file 1 — Supplementary Material 1 [file 12934_2026_2928_MOESM1_ESM.docx]

**Supplementary file**


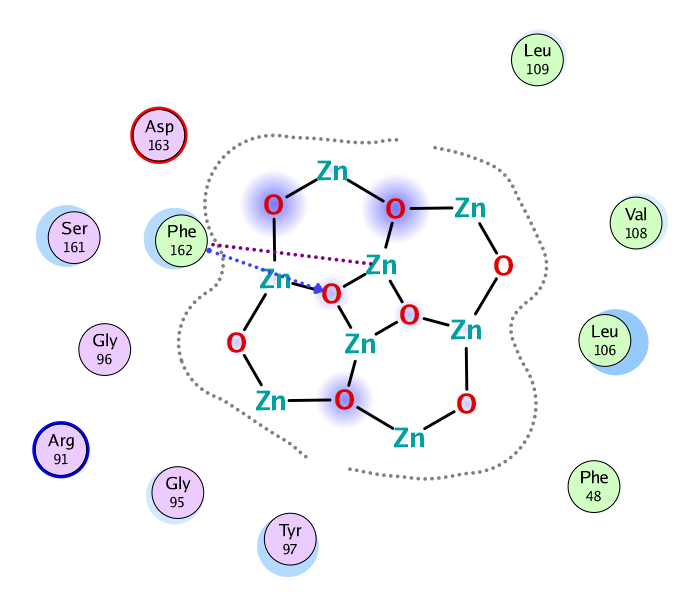


**Fig. S1** 2D diagram shows the interaction between ZnO NPs and active sites of 3T88 protein.

| 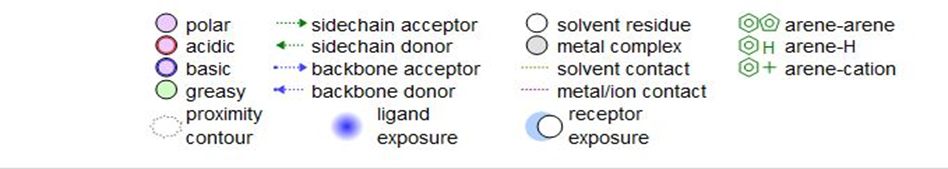 |
| --- |
| The representative key for the types of interaction between Compounds and protein receptors |


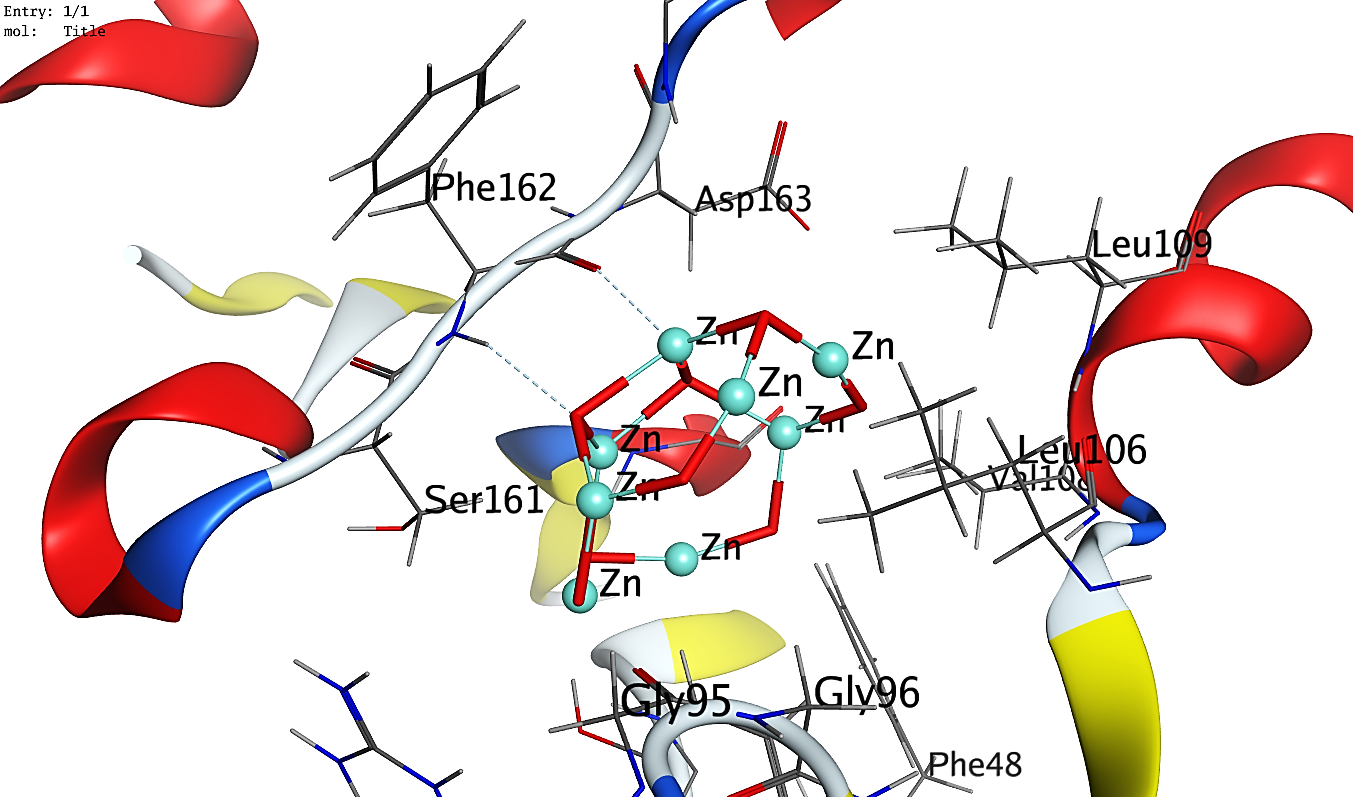


**Fig. S2** 3D diagram shows the interaction between ZnO NPs and active sites of 3T88 protein.


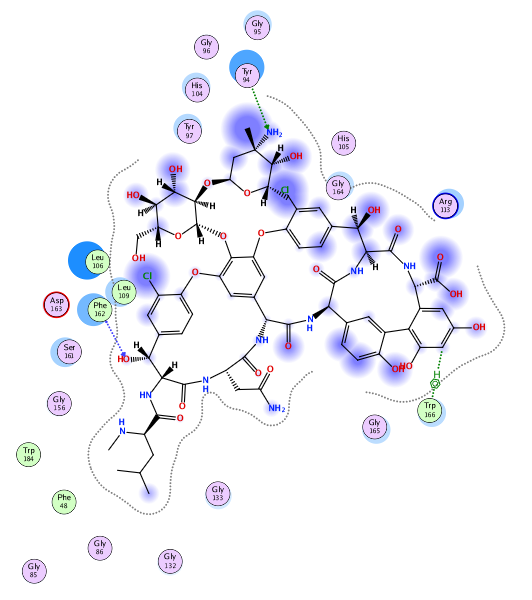


**Fig. S3** 2D diagram shows the interaction between VA and active sites of 3T88 protein.

| 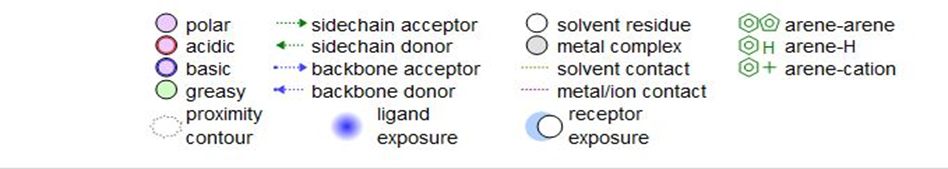 |
| --- |
| The representative key for the types of interaction between Compounds and protein receptors |


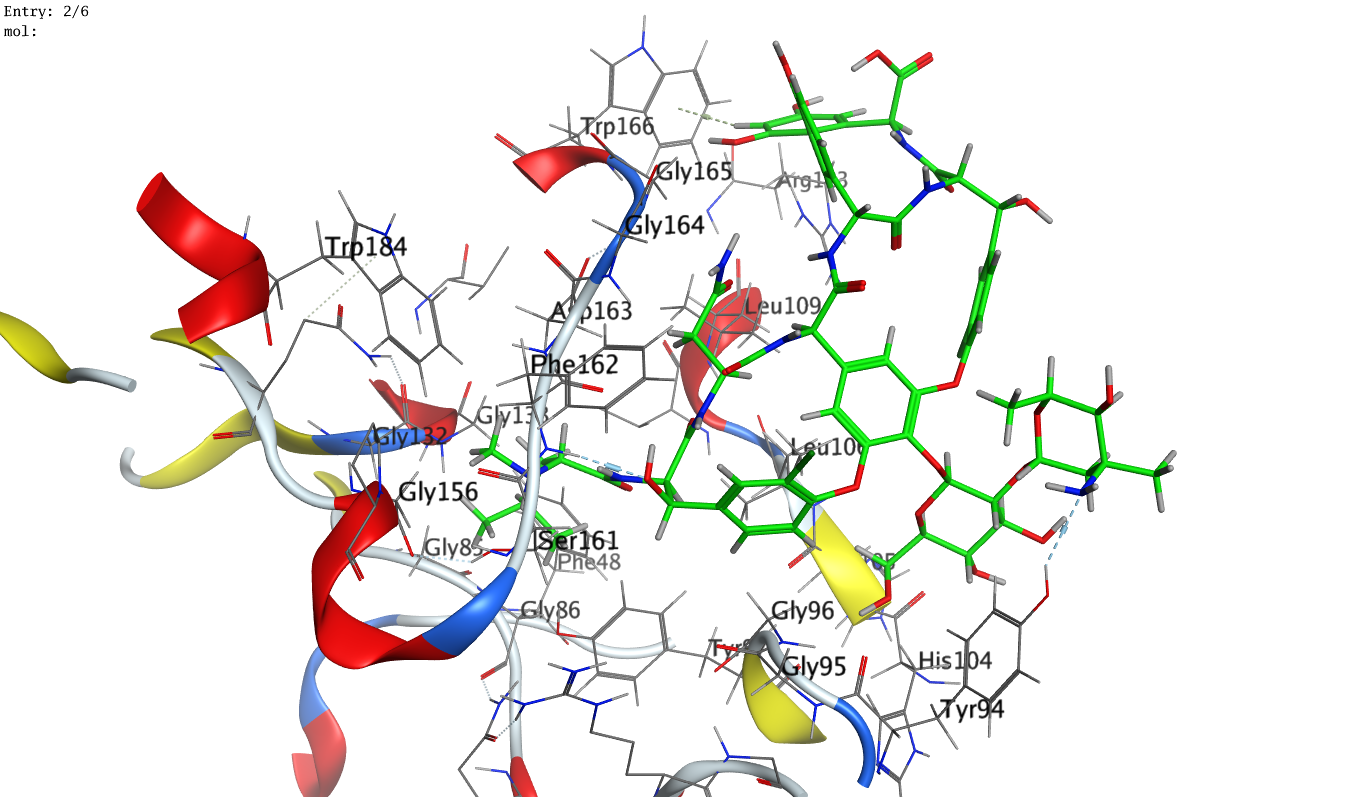


**Fig. S4** 3D diagram shows the interaction between VA and active sites of 3T88 protein.


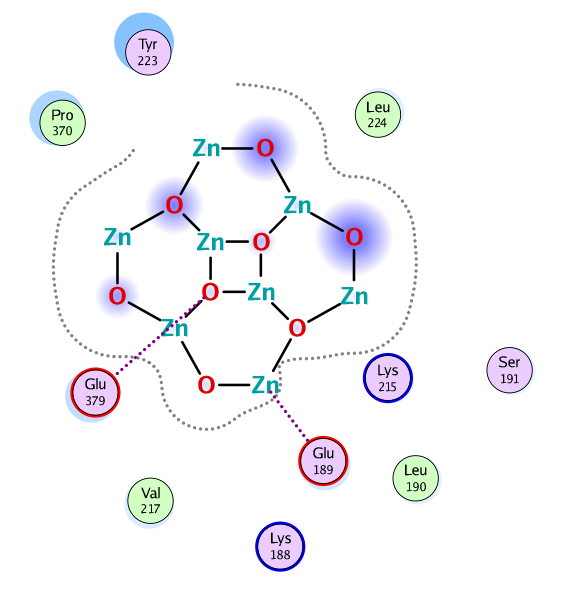


**Fig. S5** 2D diagram shows the interaction between ZnO NPs and active sites of 4DKI protein.

| 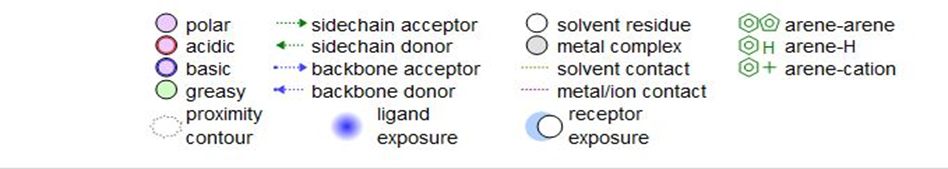 |
| --- |
| The representative key for the types of interaction between Compounds and protein receptors |


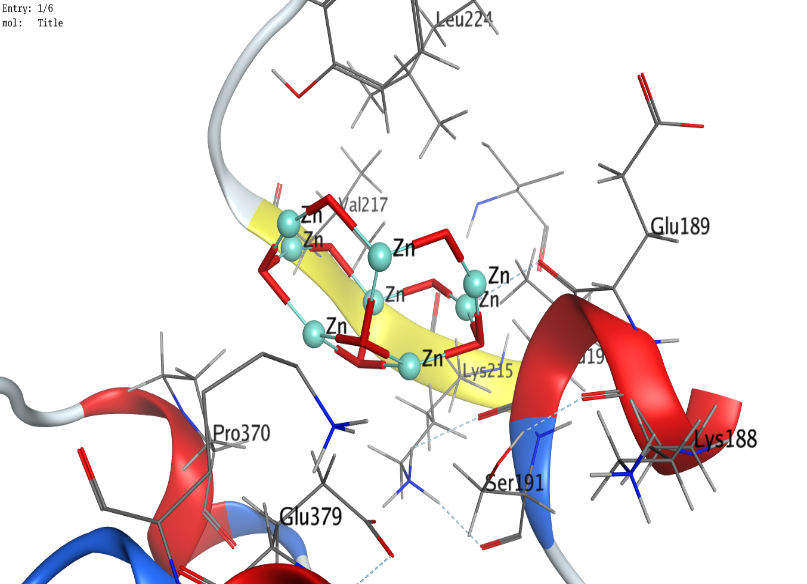


**Fig. S6** 3D diagram shows the interaction between ZnO NPs and active sites of 4DKI protein.


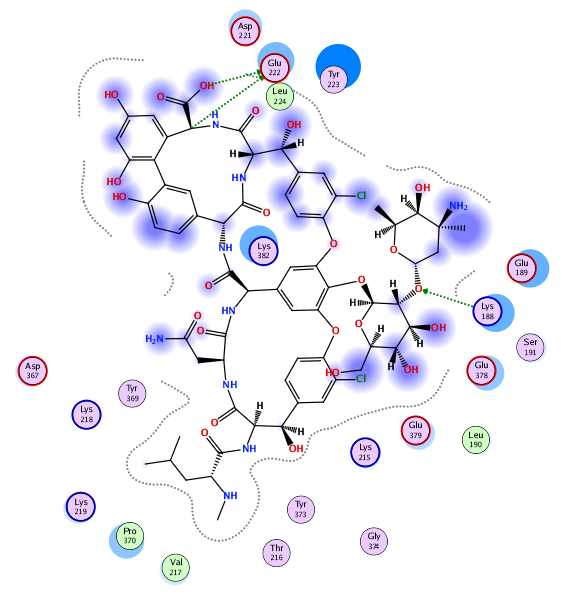


**Fig. S7** 2D diagram shows the interaction between VA and active sites of 4DKI protein.

| 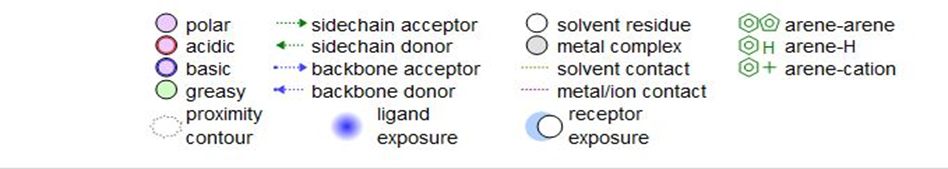 |
| --- |
| The representative key for the types of interaction between Compounds and protein receptors |


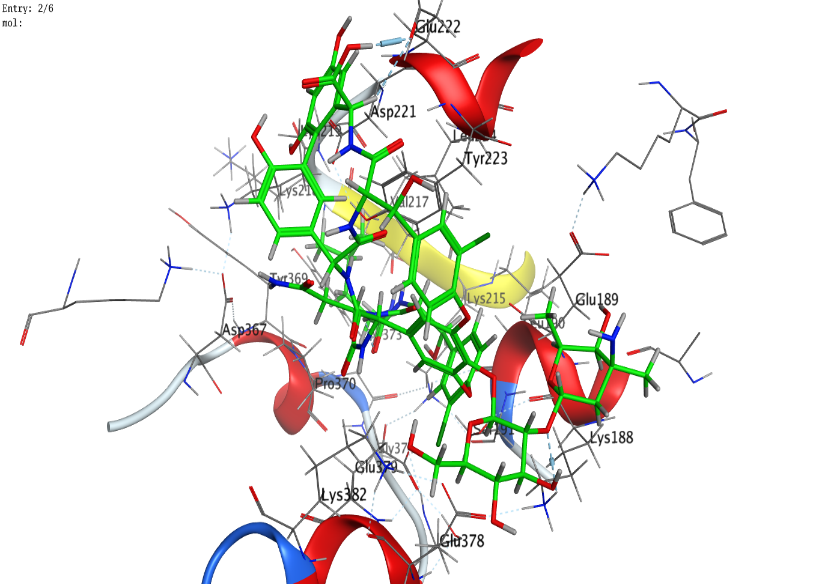


**Fig. S8** 3D diagram shows the interaction between VA and active sites of 4DKI protein.
